# Microphase separation of living cells

**DOI:** 10.1038/s41467-023-36395-2

**Published:** 2023-02-13

**Authors:** A. Carrère, J. d’Alessandro, O. Cochet-Escartin, J. Hesnard, N. Ghazi, C. Rivière, C. Anjard, F. Detcheverry, J.-P. Rieu

**Affiliations:** University of Lyon, Université Claude Bernard Lyon 1, CNRS, Institut Lumière Matière, F-69622 Villeurbanne, France

**Keywords:** Biological physics, Cellular motility

## Abstract

Self-organization of cells is central to a variety of biological systems and physical concepts of condensed matter have proven instrumental in deciphering some of their properties. Here we show that microphase separation, long studied in polymeric materials and other inert systems, has a natural counterpart in living cells. When placed below a millimetric film of liquid nutritive medium, a quasi two-dimensional, high-density population of *Dictyostelium discoideum* cells spontaneously assembles into compact domains. Their typical size of 100 μm is governed by a balance between competing interactions: an adhesion acting as a short-range attraction and promoting aggregation, and an effective long-range repulsion stemming from aerotaxis in near anoxic condition. Experimental data, a simple model and cell-based simulations all support this scenario. Our findings establish a generic mechanism for self-organization of living cells and highlight oxygen regulation as an emergent organizing principle for biological matter.

## Abstract (web summary)

In this work the authors report a quasi two-dimensional population of living cells that can spontaneously self-assemble into finite-sized domains, an analogue of the microphase separation known in inert matter.

## Introduction

It is not uncommon for physical concepts to migrate to the biological realm^1,2^. Even though cells are governed by specific genetic programming, their collective behavior shares some common generic features with non-living systems. States of matter, such as solid, liquid, gas phases originally defined for molecular components, are now employed to characterize assemblies of cells^3^. Extended analogies with liquids have illuminated the properties of tissues, whose surface tension induces cell sorting, shapes of minimal area, spreading and dewetting^1,4,5^. A variety of out-of-equilibrium processes have also proven useful: examples range from the glass and jamming transitions for epithelia^6–8^ to the directional solidification model for the growing yeast dynamics^9^ or the diffusion-limited aggregation for fractal-like bacterial colonies^10^. Over the years, soft condensed matter ideas have found fruitful applications in cell assemblies.

One soft matter phenomenon that has attracted considerable attention for half a century is micro-phase separation, a process leading to spontaneous formation of equilibrium domains with finite length scale. In the most prominent instance of diblock copolymers, separation is driven by the repulsion between two chemically different blocks but is counteracted by chain connectivity and entropy^11^. The balance between those competing trends results in ordered morphologies with a thermodynamically preferred domain size^12,13^. The microphase separation of polymers is in fact representative of a generic scenario for pattern formation: modulated phases induced by competing interactions, usually a short-range attraction opposed by a long-range repulsion^14^. This mechanism was recognized in several physical systems, including Langmuir monolayers^15^, magnetic films^16^, superconductors^17^, liquid crystals^18^ and clusters of proteins or colloids^19,20^ A related class of phenomena recently uncovered is the formation of biomolecular condensates, whether micron-scale compartments in eukaryotic cells^21^ or euchromatin domains in the nucleus^22^. All instances of microphase separation identified so far involve subcellular processes or inert matter. To our knowledge, it has never been observed in populations of cells.

We report here how living cells can self-organize in reversible finite-size domains, a bona fide analog of the microphase separation in inert matter. Cell-cell adhesion acts as a short-range attraction promoting aggregation but is counteracted by oxygen depletion and aerotaxis, whose combination induces an effective long-range repulsion. As a consequence, the domain size is set by a delicate balance between cell density and oxygen availability. An analytical model and microscopic cell simulations both corroborate this simple picture. Our findings add to the mounting evidence that oxygen regulation may govern spatial patterning in various contexts, from morphogenesis of eukaryotes to tumor escape and growth^23–25^.

## Results

### Experiments

**Fig. 1:**
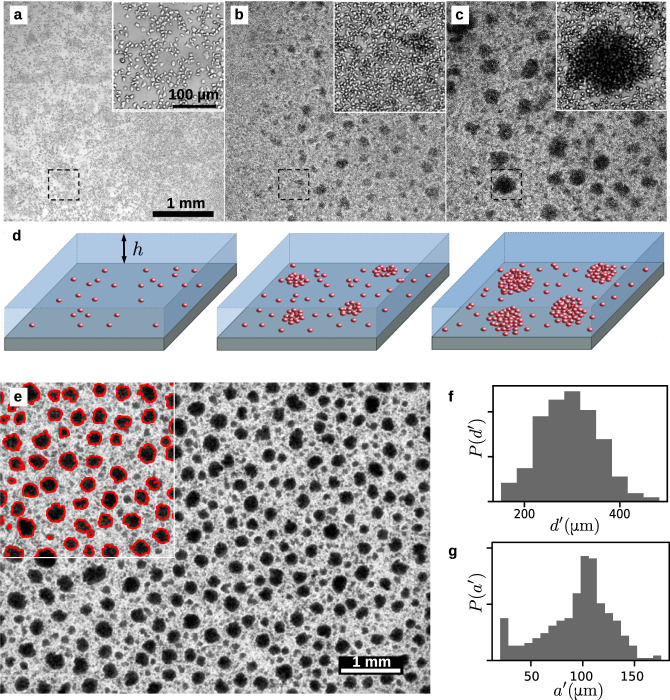
Microphase separation in a population of *Dictyostelium discoideum* cells. **a**–**c** Brightfield images of the growth and aggregation process in the course of time. Each inset is a close-up of the dashed box. The liquid film height is *h* = 1.5 mm. This experiment was repeated independently dozens of times with similar results. **a** *t* = 24 h: the cell population has grown but remains rather homogeneous. **b** *t* = 48 h: while still dividing, cells gather in small growing aggregates. **c** *t* = 110 h: the aggregates have reached a steady state. **d** Sketch of the cell arrangement in the various stages. **e** Aggregates in steady state. Large field of view (8.2 × 6.1 mm^2^) brightfield image. Here *h* = 0.85 mm. The inset shows the contour of detected aggregates. **f**, **g** Probability distribution for the inter-aggregate distance ${d}^{{\prime} }$ and the aggregate radius ${a}^{{\prime} }$ computed from (**e**).

Cells of *Dictyostelium discoideum* (*Dd*) were seeded on a substrate submerged with a film of nutrient culture medium (Fig. 1a–d) and imaged by transmission microscopy for several days (“Methods”). As their number increases through division, their spatial distribution becomes heterogeneous: cells gather in aggregates—locally denser regions—which grow by addition from the surroundings or merging with neighboring aggregates (Fig. 1b, c and Supplementary Movie 1). The steady state eventually reached (Supplementary Fig. 1) is the focus of this work.

The striking observation is the self-organization of cells in compact, roughly circular domains with a finite length scale (Fig. 1e). Dark regions, indicative of packed, multi-layered cell assemblies, exhibit both a typical size *a* and a typical center-to-center distance *d*. In Fig. 1f, g, one finds *a* = 100 ± 15 μm and *d* = 300 ± 80 μm and an aggregate thickness that usually remains below 40 μm (Supplementary Fig. 3). The aggregates stand out on a light gray background of lower density with nearly confluent cells (Fig. 1c, d insets and Supplementary Movie 2) and they are cohesive as they cannot be simply dissociated by gentle pipetting. If the calcium-dependent cell–cell adhesion system is disabled, the domains dissociate (Section B of Supplementary Information and Supplementary Movie 3), proving that aggregates involve adhesion between cells. Remarkably, the self-organized state is not frozen (Supplementary Fig. 2 and Supplementary Movie 4): aggregates remain mobile over days and though their random motion may bring them in close vicinity, they do not necessarily merge but may avoid each other, suggesting that further growth is not limited by cell dynamics but prevented by an intrinsic repulsion.

**Fig. 2:**
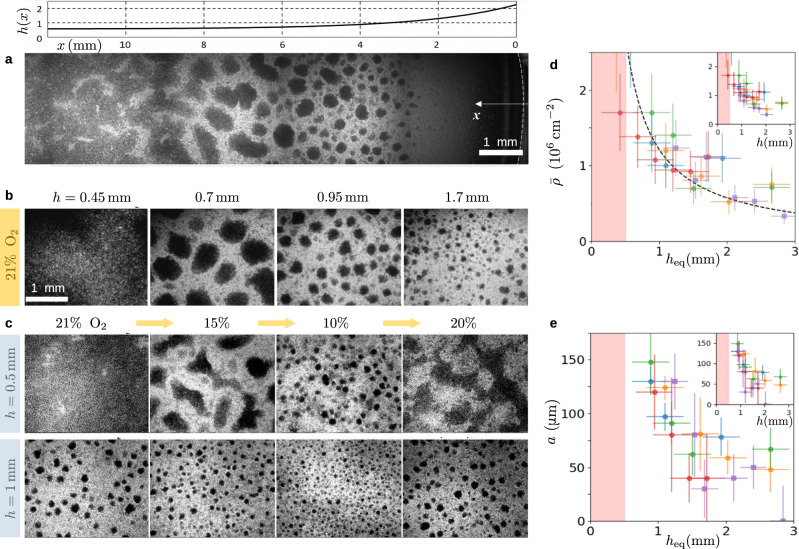
The size of aggregates is controlled by oxygen availability. **a** Domains below the meniscus at the dish border. The mosaic image was obtained by stitching confocal tile scans. *h*(*x*) is the height profile, with *x* the distance from the dish wall. This experiment was repeated independently once with similar results. **b** Influence of medium height *h* on the domain size in a normal atmosphere. This experiment was repeated independently three times with similar results. **c** Effect of a step change in the oxygen content of atmosphere while keeping *h* fixed. Each step lasts at least 6.6 h. This experiment was repeated independently twice with similar results. **d**, **e** Average (projected) cell density $\bar{\rho }$ and most probable aggregate radius *a* as a function of equivalent height ${h}_{{{{{{{{\rm{eq}}}}}}}}}\equiv h{c}_{{{{{{{{\rm{s}}}}}}}}}/{c}_{{{{{{{{\rm{s}}}}}}}}}^{{\prime} }$ (main panels) or medium height *h* (insets). Colors indicate measurements from distinct samples. Circles and squares indicate oxygen partial pressure of 21% and 15%, respectively. In panel (**d**), error bars represent the counting error (see “Methods”) and the dotted line is the prediction of Eq. (1). In panel (**e**), error bars represent the width at half maximum of the size distribution $P({a}^{{\prime} })$. Information on the numbers of aggregates considered for each point is given in Supplementary Table 1. The pink area indicates the region where a single continuous aggregate forms.

The size of self-organized domains ranges from a few dozens to hundreds of micrometers and strongly depends on the liquid height above cells. A first hint is given by cell assembly below a meniscus (Fig. 2a). In the dish center, where the film is thinnest, an almost continuous domain is seen. However, as the liquid height increases upon approaching the wall, the giant domain is replaced by finite aggregates whose size decreases until complete disappearance. In subsequent experiments (Fig. 2b), cells were placed in distinct wells with different liquid heights. Here again, the thicker the liquid film, the smaller the aggregates.

Because the substrate is impermeable and the liquid is at rest, the amount of oxygen available for cell consumption is governed by the film height *h*. Assuming a purely diffusive transport, the maximum flux of oxygen is *j*_m_(*h*) = *Dc*_s_/*h*, with *D* the diffusion coefficient and *c*_s_ the concentration at saturation which applies at the air-water interface. Using an atmosphere with controlled oxygen level (“Methods”), we changed *c*_s_ to a value ${c}_{{{{{{{{\rm{s}}}}}}}}}^{{\prime} }$ and observed that cell patterns were quite similar when the maximal flux *j*_m_ or the equivalent height ${h}_{{{{{{{{\rm{eq}}}}}}}}}\equiv h{c}_{{{{{{{{\rm{s}}}}}}}}}/{c}_{{{{{{{{\rm{s}}}}}}}}}^{{\prime} }$ was the same, which suggests that the domain size depends primarily on the available flux of oxygen. To ascertain this point, aggregates having reached a steady state were submitted to a step change of atmosphere, a process equivalent to changing *h* but without inducing any disturbance on the cells or liquid above. The effect on aggregates is immediate: domains expand in enriched atmosphere (Supplementary Figs. 4–5) and shrink in deprived atmosphere but recover their initial size upon going back to normal atmosphere (Fig. 2c and Supplementary Movie 5). Taken together, those observations demonstrate that oxygen availability, as quantified by *h*_eq_, is the key factor controlling the aggregate size.

To characterize our system, we obtain from direct measurements the medium saturation concentration *c*_s_ = 250 ± 20 μM and the cell consumption rate *q* = 4.2 ± 0.8 10^−17^ mol s^−1^ (Supplementary Fig. 6). The average cell (projected) density $\bar{\rho }$ was found to depend on *h*_eq_: the thicker the film, the lower the number of cells (Fig. 2d). It is known that cell division is strongly suppressed in anoxic condition^26,27^. Assuming that it stops entirely below a threshold ${c}_{{{{{{{{\rm{div}}}}}}}}}$, the density under a film of thickness *h* is

1 $${\bar{\rho }}_{\exp }(h)=\frac{{\xi }_{{{{{{{{\rm{div}}}}}}}}}D{c}_{{{{{{{{\rm{s}}}}}}}}}}{qh},\quad {\xi }_{{{{{{{{\rm{div}}}}}}}}}\equiv 1-\frac{{c}_{{{{{{{{\rm{div}}}}}}}}}}{{c}_{{{{{{{{\rm{s}}}}}}}}}},$$

where the literature and our own measurements (Supplementary Fig. 7) suggests ${\xi }_{{{{{{{{\rm{div}}}}}}}}}$ to be a few percent below unity. Taking ${\xi }_{{{{{{{{\rm{div}}}}}}}}}=0.95$ and using *D* = 2 10^−5^ cm^2^ s^−1 ^^28^, Eq. (1) yields $\bar{\rho }=1.13/h$, with $\bar{\rho }$ in 10^6^ cm^−2^ and *h* in mm, which is compatible with experimental data (Fig. 2d). The main observable characterizing the microphase separation is the typical aggregate size *a*, which depends on the equivalent height (Fig. 2e). For *h*_eq_ below 0.5 mm, the covering is continuous, corresponding to a formally infinite size (left side of Fig. 2a, b). When *h*_eq_ ≃ 1 mm, the largest aggregates that can be defined unambiguously are 150 μm in radius. For thicker films with *h*_eq_ = 2–3 mm, the aggregates are smaller, with radius around 50 μm. We now turn to the understanding of these experimental facts.

### A minimalistic model

We present a parsimonious approach which captures the main trends of experiments by focusing exclusively on the oxygen distribution. The model considers a fixed number of cells—there is no cell division—and involves only two basic assumptions: (i) cells spontaneously gather into aggregates with (projected) cell density *ρ*_a_, (ii) aggregates grow in size until the minimal oxygen concentration above them reaches a critical value $\hat{c}$. The former assumption is justified by cell–cell adhesion, a short-range attraction that promotes aggregation, whereas the latter acts a long-range repulsion, because oxygen depletion is stronger in larger aggregates. From this competition, a preferred domain size results. The microscopic mechanisms underlying such behavior need not be specified at this point.

**Fig. 3:**
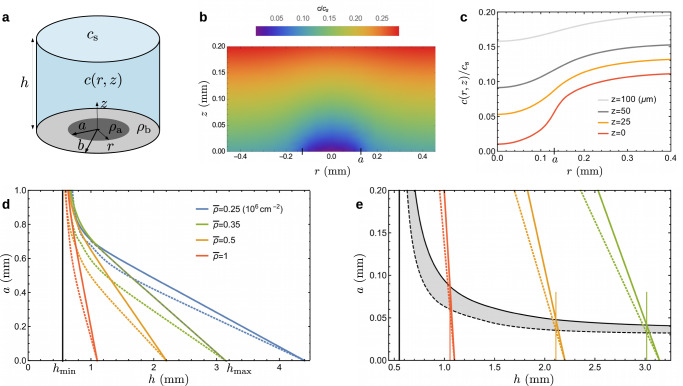
A simple model captures the domain size. **a** In the geometry considered, the aggregate and background have, respectively, radius *a* and *b*, and cell density *ρ*_a_ and *ρ*_b_, while the mean cell density $\bar{\rho }$ is fixed. The aggregate surface fraction is *ϕ* ≡ *a*^2^/*b*^2^, with maximal value ${\phi }_{\max }=\bar{\rho }/{\rho }_{{{{{{{{\rm{a}}}}}}}}}$. **b**, **c** Oxygen concentration field *c* (*r*, *z*) around an aggregate. Here $\bar{\rho }=1{0}^{6}$ cm^−2^, *h* = 1 mm, $\phi=0.16\,{\phi }_{\max }$ and the size domain is *a* = 130 μm. The minimal concentration, reached at the origin, is the target concentration $\hat{c}$, i.e., $c(0,0)/{c}_{{{{{{{{\rm{s}}}}}}}}}=\hat{c}/{c}_{{{{{{{{\rm{s}}}}}}}}}=0.01$. **d** Aggregate size $a(\bar{\rho },h,\phi )$ predicted when $\bar{\rho }$ and *h* are independent parameters, obtained from numerically solving Eq. (2). The continuous and dashed lines correspond to surface fraction $\phi={\phi }_{\max }$ and $0.16\,{\phi }_{\max }$, respectively. **e** Aggregate size *a*(*h*, *ϕ*) predicted when accounting for the thickness dependence of mean cell density $\bar{\rho }$. The black lines are solutions of Eq. (4). Graphically, they correspond to the intersection between the colored oblique lines, reproduced from panel (**d**), and the vertical line at a film height *h* solution of ${\bar{\rho }}_{\exp }(h)=\bar{\rho }$. As above, $\phi={\phi }_{\max }$ and $0.16\,{\phi }_{\max }$ for solid and dashed lines, respectively. The shaded area shows the aggregate size expected in experiments.

Our model defines a pure diffusion problem, which in a simplified geometry of disk-like aggregates (Fig. 3a), can be solved analytically (Section E of Supplementary Information). The predicted aggregate radius *a* is such that the minimal concentration, attained at the aggregate center (Fig. 3b, c), reaches the critical value $\hat{c}$. Given an average cell density $\bar{\rho }$, an aggregate surface fraction *ϕ* and a height *h*, this condition yields

2 $$\frac{a}{h}\,\psi \left(\sqrt{\phi },\frac{h}{a}\sqrt{\phi }\right)=\frac{\xi {j}_{{{{{{{{\rm{m}}}}}}}}}(h)/q-\bar{\rho }}{{\rho }_{{{{{{{{\rm{a}}}}}}}}}-\bar{\rho }},\qquad \xi \equiv 1-\frac{\hat{c}}{{c}_{{{{{{{{\rm{s}}}}}}}}}},$$

with *ψ* a known function. As visible in Fig. 3d, the existence of aggregates is limited to a range of film thickness $[{h}_{\min },{h}_{\max }]$ with

3 $${h}_{\min }=\frac{\xi D{c}_{{{{{{{{\rm{s}}}}}}}}}}{q{\rho }_{{{{{{{{\rm{a}}}}}}}}}},\qquad {h}_{\max }=\frac{\xi D{c}_{{{{{{{{\rm{s}}}}}}}}}}{q\bar{\rho }}.$$

At both ends of this interval, the system is homogeneous. For $h={h}_{\min }$, the film is so thin that even with an infinite aggregate, the concentration $\hat{c}$ is nowhere attained. For $h={h}_{\max }$, even an aggregate of vanishing size makes the minimum concentration drop below $\hat{c}$. Near ${h}_{\max }$ where the aggregate size *a* is small, the solution of Eq. (2) can be expanded as $a=\alpha ({h}_{\max }-h)$, with −*α* the slope and ${\alpha }^{-1}\equiv \psi (\sqrt{\phi },\infty )\left[{\rho }_{{{{{{{{\rm{a}}}}}}}}}/\bar{\rho }-1\right]$. Such a linear approximation is actually excellent in a wide range of height (Fig. 3d) and the intersection with the lower boundary $h={h}_{\min }$ defines a characteristic aggregate size $a\simeq {h}_{\min }/\psi (\sqrt{\phi },\infty )$, where $\psi (\sqrt{\phi },\infty )$ remains of order unity.

For a given mean cell density, the predicted aggregate size is strongly dependent on the film height, especially for the densest systems (Fig. 3d). As detailed in the Section E of Supplementary Information, we fix *ρ*_a_ = 2 10^6^ cm^−2^ and $\hat{c}/{c}_{{{{{{{{\rm{s}}}}}}}}}=0.01$. The only free parameter is the aggregate surface fraction *ϕ*. We consider both its maximum ${\phi }_{\max }=\bar{\rho }/{\rho }_{{{{{{{{\rm{a}}}}}}}}}$, reached when the background is empty of cells, and a much lower value $0.16\,{\phi }_{\max }$. In spite of this sixfold variation, the choice of *ϕ* has only a weak influence on the results. Given this parameter set, the characteristic size $a\simeq {h}_{\min }$ is at least half a millimeter, several times above the experimental sizes which remain below 200 μm. The reason is that $\bar{\rho }$ and *h* were so far treated as independent, whereas they are not in experiments.

We therefore account for the dependence of mean cell density with film height. Combining Eq. (1) for ${\bar{\rho }}_{\exp }(h)$ and Eq. (2) gives

4 $$a\,\psi \left(\sqrt{\phi },\frac{h}{a}\sqrt{\phi }\right)=\Delta \xi {\left[\frac{\xi }{{h}_{\min }}-\frac{1}{h}\right]}^{-1},$$

where $\Delta \xi=\xi -{\xi }_{{{{{{{{\rm{div}}}}}}}}}$. A numerical solution is shown in Fig. 3e with black lines. In particular, Eq. (4) indicates that for large $h/{h}_{\min }$, the size is $a\simeq {h}_{\min }\,\Delta \xi /\xi$. The thickness dependence of cell density has thus two important implications. First, the typical aggregate size is not any more ${h}_{\min }$, which was millimetric, but a much lower value since Δ*ξ* = 0.04. For instance, when *h* = 1 mm, a size in the range 60–100 μm is predicted, which now overlaps with the experimental observations. Second, the aggregates can exist at water heights of several millimeters. Both consequences originate from the same fact: the mean density spontaneously reached by the system corresponds to a liquid height slightly below the value ${h}_{\max }$ where aggregates would disappear (Fig. 3e).

Let us briefly consider the model predictions (Fig. 3d) for a step change in atmosphere when the cell density remains constant, since cells have no time to divide. When suddenly submitted to an oxygen-deprived atmosphere, aggregates should disappear. A clear reduction is observed (Fig. 2c and Supplementary Movie 5), though not a complete disappearance that we suspect is prevented by an increasingly slow dynamics. When submitted to an enriched atmosphere, aggregates are predicted to become larger, a trend that is unmistakably observed (Supplementary Fig. 4) even if the final sizes are again difficult to ascertain. Thus, in spite of drastic approximations, our elementary model recapitulates the main features of aggregate behavior and supports the notion that oxygen regulation governs domain formation.

### Cell-based simulations

**Fig. 4:**
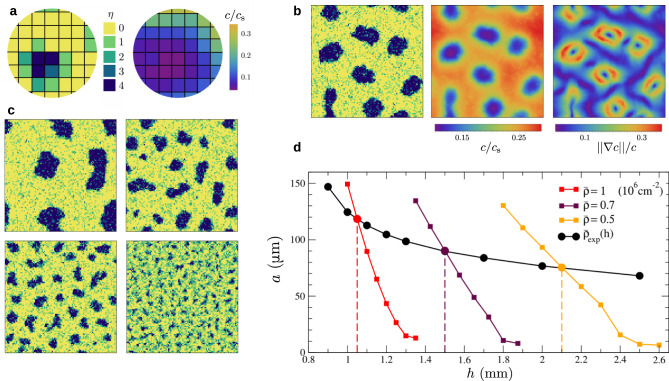
Cell-based simulations provide a microscopic view of microphase separation. **a** Schematic of the two-dimensional lattice model. Each site is occupied by a number *η* of cells, with maximum ${\eta }_{\max }=4$. The oxygen concentration field is assumed steady. Cells adhere to their neighbors, consume oxygen and become aerotactic at low concentration when *c*/*c*_s_ < *c*_aer_ = 0.1. **b** Example of aggregate. From left to right, occupation number, oxygen concentration and term ∣∣∇*c*∣∣/*c* controlling aerotaxis. The cell density is $\bar{\rho }=\,1{0}^{6}$ cm^−2^, the height *h* = 1.1 mm and the lattice size *L* = 100. **c** The aggregate size depends on film height: simulations with *h* = 1.05, 1.15, 1.2, 1.3 mm yield an aggregate size *a* = 118, 65, 43, 15 ± 5 μm, respectively. Here $\bar{\rho }={10}^{6}$ cm^−2^ and *L* = 100. **d** Typical aggregate size as a function of film height, at fixed cell density $\bar{\rho }$ (colored squares). The vertical lines indicate the film height solution of ${\bar{\rho }}_{\exp }(h)=\bar{\rho }$. Shown with black dots is the typical aggregate size expected in experiments. Lines are guides to the eye.

We now propose a microscopic description of the domain formation and illustrate how the picture of the analytic model can originate in the individual behavior of the cells. In our two-dimensional lattice model (Fig. 4a), a site can accommodate several cells, each of which obey three rules. First, cell-cell adhesion favors contact with their neighbors. Second, cells consume oxygen. Third, they exhibit aerotaxis toward oxygen-rich regions, but only in near anoxic condition when *c*/*c*_s_ < *c*_aer_ = 0.1, as demonstrated recently^29^. The number of cells is constant because division is not considered. In our simulations, the oxygen concentration field, assumed steady, is evaluated with a Green function and the cells move according to a Monte Carlo (MC) algorithm with local moves only. A realization typically involves 10^4^ cells and 10^6^–10^7^ MC steps. The model definition, numerical method and parameters are detailed in “Methods”.

The competition between adhesion and aerotaxis is sufficient to drive the formation of finite-size aggregates (Fig. 4b, c). In the absence of aerotaxis, cells form aggregates, which further grow by fusion events. Such a coarsening process would lead, albeit slowly, to a divergence of domain size. In the presence of aerotaxis, the aggregate size converges to a finite value, indicating a stationary state. The microscopic mechanism can be inferred from Fig. 4b. Cell consumption induces oxygen depletion near aggregates, generating gradients that below *c*_aer_ trigger aerotactic escape and limit further domain growth. Consistent with this picture, the domain size is controlled by film thickness *h* and mean cell density $\bar{\rho }$, as visible in Fig. 4d. A comparison with Fig. 3d reveals that qualitatively the trends in the model and simulations are identical. Aggregates appear only below a height ${h}_{\max }$ and upon decreasing *h* from this point, their size increases linearly, with a slope controlled by the density $\bar{\rho }$. The simulations thus provide a microscopic basis for the behavior postulated in the minimalistic model.

For a quantitative comparison between simulations and experiments, we fix the cell size to 10 μm and take all other parameters from their known value, except the cell consumption *q*, which is reduced by a factor 1.2 (“Methods”). Accounting for the height dependence of mean cell density, the aggregate size found in simulations is shown in Fig. 4d with the black curve. As the film height varies from 1 to 2.5 mm, the aggregate radius decreases from 120 to 70 μm, a trend that is also seen in experimental data. Besides, as in experiments, the simulated aggregates never reach a static configuration but continually deform, move and rearrange (“Methods” and Supplementary Movie 6). Such a feature is typically absent from inert systems and might be specific to microphase separation of living cells. All together, our cell-based simulations can capture the key features of aggregate behavior and confirm that oxygen-controlled domain formation can result solely from the interplay between adhesion, consumption and aerotaxis.

## Discussion

Quasi two-dimensional assemblies of *Dd* cells under liquid films can self-organize into domains of finite size because the combined effect of oxygen depletion and aerotaxis leads to an effective long-range repulsion that counteracts spontaneous aggregation. The mechanism is similar to the microphase separation of inert systems. There is, however, an interesting difference. Unlike purely two-dimensional systems^19,30,31^, the repulsion is mediated by the liquid film, whose equivalent thickness *h*_eq_ determines the range of effective interaction. Because *h*_eq_ is easily varied experimentally, it offers some control on the domain size, which can be changed dynamically. This is in stark contrast with block copolymers where the long-range repulsion is primarily governed by chain properties and is thus fixed once and for all^13^.

Since domain formation in living cells has been observed in the past, it is worth pointing out how the involved phenomena, though superficially resembling, are actually different from the microphase separation reported here. The bacterial chemotactic patterns of ref. ^32^ exhibit finite-size spots but in contrast with our aggregates, they are irreversibly “frozen in” and made of immobile bacteria. In the microcolonies of *Neisseria gonorrhoeae*^33,34^, where coarsening would eventually lead to a giant monodomain, the finite size is a consequence of kinetic slowdown and not the result of competing interactions. The stripe patterns of bacteria with density-dependent motility^35^ involves a continuously expanding population, not a fixed number of cells. Finally, the classical aggregation of *Dd*, formalized in the chemotactic collapse of the Keller-Segel model^36^, results in a single millimetric immobile mound^37^, clearly distinct from our smaller and dynamic aggregates. On the theoretical side, we note that Turing patterns^38,39^ often imply the constant creation and destruction of species. In contrast, microphase separation involves only the rearrangement of cells whose number is locally conserved.

The microphase separation of living cells opens questions and perspectives. First, we have focused on the domain size in steady state but many aspects call for in-depth exploration: the kinetics of domain formation and the interplay with cell division, the tridimensional structure of aggregates and their random motion, mode of migration and chemotactic ability^40,41^. Second, an understanding of the biological mechanism underlying cell behaviors is needed. Third, we anticipate that the microphase separation demonstrated with *Dd* cells has a wider relevance because adhesion and aerotaxis are both widespread throughout the living world, from bacteria to higher eukaryotic cells^42,43^.

We conclude on the broader significance of our findings in the context of biological patterning and morphogenesis. Embryonic development was long attributed to an inductive process governed by exogenous signals such as maternal chemical gradients or morphogens^44^. In contrast, a recent line of work with embryoid models emphasizes the evidence for endogenous self-organization^45^. The two mechanisms may actually act concurrently, a combination termed guided self-organization. The oxygen-controlled microphase separation found here offers a striking instance of such a hybrid process. No pattern is imposed from outside but the availability of oxygen, that may be controlled externally, dictates whether cells aggregate or not and which domain size is ultimately selected. Interestingly, a related approach of “directed assembly” has been exploited for block copolymers^46^. Guided self-organization is a thus generic mechanism relevant both in technological applications and in the natural realm. Finally, multicellular aerobic life is inevitably constrained by the fact that diffusion is slow over large distances^47,48^. While the genesis of multicellularity remains debated^49^, one may wonder whether microphase separation is involved and possibly reassess the role of oxygen in pattern formation and life evolution. We expect physical concepts to be instrumental in this endeavor.

## Methods

### Experiments

#### Cell preparation and live cell imaging

AX2 axenic cells of *Dictyostelium discoideum* were grown in HL5 medium with glucose (Formedium, Norfolk, UK) at 22 °C in tubes with shaking conditions (180 rpm for oxygenation). Exponentially growing cells were harvested, counted and deposited in 6-well plates, with a initial surface cell density *ρ*_0_ between 5 × 10^3^ and 2 × 10^5^ cells/cm^2^. Cells were submerged below liquid growth medium with height ranging from *h* = 0.5  to 3 mm. The temperature was kept constant at 22 °C.

We observed the growth and aggregation of cells under this free liquid film for days. We also performed experiments where the liquid film is topped with a thin layer of oil (low viscosity paraffin oil, ref. 294365H, VWR Chemicals). Because the product of solubility and diffusion coefficient of oxygen in oil is high, the presence of oil does not appreciably reduce the availability of oxygen and the final mean aggregate size is close (within 10%) to that observed without oil.

The growth and aggregation of *Dd* cells were observed in transmission with three types of microscope: (i) a TE2000-E inverted microscope (Nikon) controlled with Micromanager (version 2.0 gamma) and equipped with a motorized stage, a 4X Plan Fluor objective lens (Nikon) and a Zyla camera (Andor) using brightfield for most of the timelapse experiments lasting up to several days (Figs. 1a–c, 2b, c, Supplementary Figs. 4–5, and Supplementary Movies 1, 3, and 5), (ii) a binocular MZ16 (Leica) controlled with LAS X software (version 3.4.2 Leica) and equipped with a TL3000 Ergo transmitted light base (Leica) operated in the one-sided darkfield illumination mode and a Leica LC/DMC camera (Leica) for large field experiments (Fig. 1e, Supplementary Figs. 1–2 and Supplementary Movie 4) and finally (iii) a confocal microscope (Leica SP5) controlled with the LAS software (version 3.4 Leica) and equipped with a 10X objective lens for larger magnification experiments (Supplementary Fig. 3A–C and Supplementary Movie 2) and with a Tile Scan to create large field reconstituted mosaic images (Fig. 2a).

#### Atmosphere with controlled oxygen level

In several experiments (Fig. 2b, c, Supplementary Figs. 4–5), we varied the oxygen concentration in the atmosphere surrounding the liquid film. Plates were placed in a home-made environmental chamber fitting our microscope stage with a pair of top and bottom glass windows. A mixture with pure N_2_ (0% O_2_) and air (21% O_2_) was prepared in a gas mixer (Oko-lab 2GF-MIXER, Pozzuoli, Italia) and injected continuously in the chambers at about 300 mL/min, so that the oxygen level in the atmosphere can be maintained at a prescribed value.

#### Medium height profile

The medium height *h* in dish center was estimated as a function of time by dividing the corrected volume by the dish area, typically 9.6 cm^2^ in 6-well plates. The corrected volume is the initial culture medium volume from which are subtracted the evaporated volume *V*_evap_ and the meniscus volume *V*_menis_. The evaporated volume *V*_evap_ was deduced by measuring the remaining sample volume in a precise serological pipet at the end of each experiment. It was extrapolated to any time assuming a constant evaporation rate. From the height profile in the small slope approximation $h(x)=h+\delta h(0)\exp (-x/{L}_{{{{{{{{\rm{c}}}}}}}}})$, the meniscus volume was computed as ${V}_{{{{{{{{\rm{menis}}}}}}}}}={{{{{{{\mathcal{P}}}}}}}}\delta h(0)$. Here ${L}_{{{{{{{{\rm{c}}}}}}}}}=\sqrt{\gamma /\rho g}$ is the capillary length^50^, ${{{{{{{\mathcal{P}}}}}}}}$ is the dish perimeter, $\delta h(0)={L}_{{{{{{{{\rm{c}}}}}}}}}\sqrt{2(1-\sin \theta )}$ is the meniscus height at the dish wall (*x* = 0) relative to the medium height *h* in the center and *θ* is the contact angle. The same small slope approximation was used to plot the meniscus profile of Fig. 2a. The surface tension, measured with a Langmuir balance (NIMA, England), is *γ* = 55 mN/m. The contact angle value *θ* = 48 ^∘^ was obtained for a 3-day sample and used for the remainder of the study. Its value was periodically verified by taking side view images of the meniscus.

#### Image analysis

*Aggregates*. A home-made algorithm in Python was used to detect the domains. A typical image is shown in Fig. 1e of the main text, where the boundary of identified aggregates is drawn in red. We note that some clumps of cells, with small or moderate size and characterized by intermediate gray level, may or may not be identified as aggregates. The domains with large size, which are most often very dark, are detected unambiguously, i.e., they are not sensitive to the precise parameters used in the algorithm. They thus define a robust population of domains, which is the focus of our study.

Once aggregates are detected, we compute their area ${{{{{{{\mathcal{A}}}}}}}}$, their equivalent radius ${a}^{{\prime} }=\sqrt{{{{{{{{\mathcal{A}}}}}}}}/\pi }$ and identify the typical radius *a* from the maximum in the distribution $P({a}^{{\prime} })$. We choose the maximum and not the mean of the distribution, because the former is independent of values found for small aggregates whereas the latter is not. For the same reason, we use the width at half maximum to characterize the dispersion around the most probable value. Finally, we compute for each aggregate the distance ${d}^{{\prime} }$ to the nearest neighbor. From the maximum in the distribution $P({d}^{{\prime} })$, we obtain a typical inter-aggregate distance *d*, and from the width at half maximum, the characteristic dispersion.

#### Cell density

*Average cell density*. A the end of each experiment, cells were detached by thorough pipetting, the cell suspension in liquid medium was immediately collected with a serological pipet and part of it was used in a hemocytometer to obtain the average (projected) cell density $\bar{\rho }$. The counting was repeated twice with two different cell suspension sample volumes to estimate the counting error and the corresponding standard deviation.

*Background cell density*. The background is defined as the area outside identified aggregates. The cell density *ρ*_b_ in the background can be determined by automatic detection of individual cells on images using the Find-Maxima plugin in ImageJ.

### Simulations of a cell-based model

#### Model definition and parameters

We introduce a minimal model with two basic ingredients. The first is adhesion between cells that are in contact, which favors the formation of aggregates. The second is aerotaxis, which drives motion along the gradient of oxygen and opposes the growth of large domains.

The model is lattice-based and represents each cell individually. A cell occupies a single site of a two-dimensional square lattice. To account in an effective manner for the three-dimensional structure of the aggregates, which may have several layers, a site can accommodate several cells. The number *η*_*m*_ of cells at site *m* is limited to a maximum value ${\eta }_{\max }$, which reflects the limited height of aggregates. We define the neighbors of a site as the eight sites surrounding it. When located on the same site or on neighboring sites, two cells are in contact and each contact contributes an energy −*ε*. The adhesion energy of a cell at site *m* is therefore

5 $${E}_{m}^{{{{{{{{\rm{adh}}}}}}}}}=-\varepsilon \left[{\eta }_{m}-1+\mathop{\sum}\limits_{{m}^{{\prime} }}{\eta }_{{m}^{{\prime} }}\right],$$

where the sum runs over all neighboring sites ${m}^{{\prime} }$.

Each cell *l* consumes oxygen with an individual rate *Q*_*l*_ fixed by the local concentration *c*,

6 $${Q}_{l}={q}_{\max }\tanh \left(\frac{c}{{c}_{{{{{{{{\rm{csm}}}}}}}}}}\right)\equiv {q}_{\max }\,{q}_{l}.$$

where ${q}_{\max }$ is the maximal consumption, *q*_*l*_ a rescaled consumption and *c*_csm_ a characteristic concentration. The cell consumption is almost constant above *c*_csm_ and approximately linear in *c* below *c*_csm_. The liquid film is not represented explicitly. Instead, the oxygen concentration field is evaluated from the cell positions by assuming it has reached a steady state. The method to do so relies on a Green function and is detailed below.

Each cell is endowed with aerotactic behavior, which sets in below a characteristic concentration *c*_aer_. We assume logarithmic sensing^51^ and a drift velocity ${v}_{{{{{{{{\rm{aer}}}}}}}}} \sim \nabla (\ln c)$. To a move from site *m* to a neighboring site ${m}^{{\prime} }$, we associate an energy

7 $$\Delta {E}_{m\to {m}^{{\prime} }}^{{{{{{{{\rm{aer}}}}}}}}}=-\chi {{{{{{{\mathcal{H}}}}}}}}[{c}_{{{{{{{{\rm{aer}}}}}}}}}-{c}_{m}]\,\frac{{(\nabla c)}_{m\to {m}^{{\prime} }}}{{c}_{m}},$$

where *χ* quantifies the strength of aerotaxis, *c*_*m*_ is the oxygen concentration at site *m*, ${{{{{{{\mathcal{H}}}}}}}}$ is a smoothed Heaviside function and ${(\nabla c)}_{m\to {m}^{{\prime} }}$ is the concentration gradient at site *m* in the direction of site ${m}^{{\prime} }$.

The motion of cells is simulated using a basic Monte Carlo (MC) algorithm. MC moves are only local, i.e., the cell move only to one of the neighboring sites. The total energy change of a move from site *m* to site ${m}^{{\prime} }$ is

8 $$\Delta {E}_{m\to {m}^{{\prime} }}=\Delta {E}_{m\to {m}^{{\prime} }}^{{{{{{{{\rm{adh}}}}}}}}}+\Delta {E}_{m\to {m}^{{\prime} }}^{{{{{{{{\rm{aer}}}}}}}}}+\Delta {E}_{m\to {m}^{{\prime} }}^{{{{{{{{\rm{occ}}}}}}}}},$$

where $\Delta {E}_{m\to {m}^{{\prime} }}^{{{{{{{{\rm{adh}}}}}}}}}$ is the variation in adhesion energy. $\Delta {E}_{m\to {m}^{{\prime} }}^{{{{{{{{\rm{occ}}}}}}}}}$ is infinite if the new site ${m}^{{\prime} }$ is already maximally occupied (${\eta }_{{m}^{{\prime} }}={\eta }_{\max }$) and zero otherwise. A proposed move is accepted according to the Metropolis criterion, that is with probability

9 $${p}_{{{{{{{{\rm{acc}}}}}}}}}=\min \left[1,\exp \left(-\frac{\Delta E}{{k}_{{{{{{{{\rm{B}}}}}}}}}T}\right)\right],$$

with *T* the temperature and *k*_B_ the Boltzmann constant. During a Monte Carlo step, each cell is subject to one proposed move.

As regards the units and parameters, the lattice spacing or cell size $\bar{b}$ is taken as unit length and we fix $\bar{b}=10\,\upmu$m. Accordingly, if each site is occupied on average by one cell, the dimensionless mean cell density is $\bar{\rho }=1$, which corresponds to a real density $\bar{\rho }={\bar{b}}^{-2}=1{0}^{6}$ cells/cm^2^. The energy unit is *k*_B_*T* and concentrations are made dimensionless with respect to the saturation value *c*_s_. The parameters of the model are listed in Table 1, together with a default value which is justified below. Simulation units are understood when no unit is specified.

**Table 1:** Units and parameters of the model

Symbol	Value	Quantity
$\bar{b}$	–	Unit length (*b* ↔ 10 μm)
*k*_B_*T*	–	Unit energy
*c*_s_	–	Unit concentration
$\bar{\rho }$	1	Mean cell density
${\eta }_{\max }$	4	Maximum occupation number
*ε*	0.15	Contact energy
*h*	1	Film height expressed in millimeter
*κ*	0.85/1.2	Field parameter as defined by Eq. (16)
*c*_csm_	0.02	Concentration around which consumption becomes non-constant (Eq. (6))
*c*_aer_	0.1	Concentration below which aerotaxis sets in Eq. (7)
*χ*	2	Aerotaxis strength (Eq. (7))
*L*	100	Lattice size
${T}_{{{{{{{{\rm{sim}}}}}}}}}$	10^6^	Simulated time in MC steps

A default value, which holds unless indicated otherwise, is also indicated.

#### Method

*Green function.* We consider a liquid film of height *h* whose top surface is in contact with air. Assume a (non-uniform) absorbing flux *j* is imposed at the bottom surface. What is the resulting oxygen concentration field in steady state? Here we give a partial response using a Green function. It is convenient to work with the shifted concentration

10 $$f(x,y,z)=1-\frac{c(x,y,z)}{{c}_{{{{{{{{\rm{s}}}}}}}}}}.$$

Choosing units here so that *h*, *D* and *c*_s_ are all unity, the equation and boundary conditions are: $\Delta f=0 \quad {{{\mbox{for}}}}\quad 0 \, < \, z \, < \,1,$ $f=0 \quad {{{\mbox{for}}}} \quad z=1$ and ${\partial }_{z}\,f=j({{{{{{{\bf{r}}}}}}}}) \quad {{{\mbox{for}}}} \quad z=0,$ with Δ the Laplacian and **r** = (*x*, *y*) the position in the plane. Taking Fourier transforms with respect to *x* and *y* variables leads to

11 $$\left(-{k}^{2}+{\partial }_{zz}^{2}\right)f({k}_{x},{k}_{y},z)=0,\qquad k\equiv \sqrt{{k}_{x}^{2}+{k}_{y}^{2}},$$

whose solution is a combination of exponential $\exp (\pm kz)$. Exploiting the boundary conditions gives

12 $$f({{{{{{{\bf{k}}}}}}}},z)=j({{{{{{{\bf{k}}}}}}}})\frac{\sinh (k(1-z))}{k\cosh (k)}.$$

Considering only the surface and putting back dimensions, we get

13 $$f({{{{{{{\bf{k}}}}}}}},z=0)=\frac{j({{{{{{{\bf{k}}}}}}}})}{D{c}_{{{{{{{{\rm{s}}}}}}}}}/h}\frac{\tanh (hk)}{hk}.$$

Note that this solution is valid only if the concentration remains positive everywhere.

Given a site **r**_*l*_ and a consumption *q*_*l*_ for each cell *l*, the local flux at a position **r** of the bottom substrate is

14 $$j({{{{{{{\bf{r}}}}}}}})=\mathop{\sum}\limits_{l}\frac{{Q}_{l}}{{\bar{b}}^{2}}\delta ({{{{{{{\bf{r}}}}}}}},{{{{{{{{\bf{r}}}}}}}}}_{l})\equiv \frac{{q}_{\max }}{{\bar{b}}^{2}}{{{{{{{\mathcal{J}}}}}}}}({{{{{{{\bf{r}}}}}}}}),$$

where *δ*(**r**, **r**_*l*_) is 1 when **r** belong to the site occupied by cell *l* and 0 otherwise, and the sum runs over all cells. The shifted concentration field is then

15 $$f({{{{{{{\bf{k}}}}}}}},z=0)={{{{{{{\mathcal{J}}}}}}}}({{{{{{{\bf{k}}}}}}}})\frac{\tanh (hk)}{hk}\times \kappa \times h[{{{{{{{\rm{mm}}}}}}}}],$$

where *h*[mm] is the film thickness expressed in millimeter for convenience and *κ* a parameter defined as

16 $$\kappa \equiv \frac{{q}_{\max }}{{\bar{b}}^{2}{c}_{{{{{{{{\rm{s}}}}}}}}}D}\times 1{0}^{-3}.$$

With *c*_s_ = 250 μM, *D* = 2 × 10^−5^ cm^2^ s^−1^ and ${q}_{\max }=4.2\times1{0}^{-17}$ mol s^−1^ (Supplementary Fig. 6), we have *κ* = 0.85, a value of order unity.

In practice, Fast Fourier transforms are used to switch between real and Fourier space representation of functions and obtain from Eq. (15) the shifted concentration at the bottom surface *f*(**r**, *z* = 0). The gradient of concentration is also computed in Fourier space.

*Concentration field and cell consumption.* The oxygen concentration field and individual cell consumptions are coupled variables. For any cell *l*, at any time, they should verify

17 $${q}_{l}={f}_{{{{{{{{\rm{csm}}}}}}}}}(c({{{{{{{{\bf{r}}}}}}}}}_{l})),$$

where the concentration *c*(**r**) depends on the whole set {*q*_*l*_} of cell consumptions. To ensure that this constraint holds, we used an iteration algorithm, which converges toward a fixed point solution of Eq. (17). We observe numerically that concentrations can be small, down to a few percent, but always remain positive as required.

In principle, the concentration field and cell consumptions should be updated each time a cell changes position. In practice however, this is exceedingly costly in computational resources as the concentration update is the limiting step in the simulation. Accordingly, we resort to a simple approximation where the field remains constant during one MC step and is updated after each MC step. Given that the MC moves considered are only local, that the oxygen concentration depends on many cells, and that aggregates evolve on time scale much longer than a single MC step, this approximation is well justified. As a side remark, we note that the transient decoupling between particles and field is similar to the methods used for simulations of block copolymers^52^.

*Aggregate size.* The size of aggregates is the main quantity of interest. In defining aggregates, only sites with occupation number *η* ⩾ 3 are considered. Using a connected component analysis, aggregates are identified and sorted in order of decreasing area ${{{{{{{\mathcal{A}}}}}}}}$, defined as the number of sites composing the aggregate. We build a representative set by including aggregates, with larger ones coming first, until the sum of their areas exceeds 90% of the total number of aggregate sites. Such a definition allows to discard the small but potentially numerous aggregates and to focus on the larger aggregates that are of interest in this work. This definition is also suitable if there is a single or only a few aggregates. Finally, we compute the mean area $\langle {{{{{{{\mathcal{A}}}}}}}}\rangle$ of the aggregates in the representative set and use the radius $a=\sqrt{\langle {{{{{{{\mathcal{A}}}}}}}}\rangle /\pi }$ as a simple measure for the typical aggregate size.

#### Simulations

*Choice of parameters*. Here we briefly explain how the parameters were chosen. First, the typical dimension of *Dictyostelium discoideum* cells is fixed to $\bar{b}=10\,\upmu$m, a convenient value that is approximately consistent with the range of cell size observed. Since the thickest aggregates reach 40 μm, as shown in Section B of Supplementary Information, the maximum occupation number is set to ${\eta }_{\max }=4$. As regards the adhesion energy *ε*, and for simulations when only adhesion is present, we note the following. For ${\eta }_{\max }\varepsilon \,\ll \,1$, aggregates are negligible in size. For ${\eta }_{\max }\varepsilon \,\gg \,1$, aggregation is irreversible: aggregates quickly form but remain frozen in size and do not coalesce further because cells are irreversibly stuck. The case ${\eta }_{\max }\varepsilon \simeq 1$ is the interesting regime: large aggregates appear and coexist with a “gas” of isolated cells, with constant exchange between them. Small *ε* values favor a dense gas and aggregates with branched, irregular shapes while larger values yield more compact shapes but a gas of vanishing density. We choose *ε* = 0.15, in *k*_B_*T* units, as a compromise that features both rather rounded aggregates and a significant gas around them.

The typical concentration below which the individual consumption of cells becomes concentration-dependent is *c*_csm_ = 0.02, which is consistent with the range given by our measurements (see Supplementary Fig. 6). Aerotactic behavior sets in below *c*_aer_ = 0.1 as found in ref. ^29^. The aerotactic strength is chosen so that when oxygen levels are everywhere below *c*_aer_, no sizeable aggregate form. A value *χ* = 2 is sufficient to enforce this requirement. Finally, the individual cell consumption was reduced by a factor 1.2, meaning that *κ* = 0.85/1.2 as indicated in Table 1. This choice allows for a quantitative comparison between simulations and experiments. Note that a reduction in consumption may be physically understood because our two-dimensional model can not accurately describe the effects within three-dimensional aggregates, such as limited oxygen availability for cells most underneath.

As a side remark, we note that our model does not account for cell division. Even though our parameters may lead to oxygen concentrations above ${c}_{{{{{{{{\rm{div}}}}}}}}}$, the number of cells remains constant. Including cell division is far from straightforward because the very low concentrations involved (below ${c}_{{{{{{{{\rm{div}}}}}}}}}$) require the constant adjustment of individual cell consumption and drastically increase the computation time. This is why we have have chosen to focus on the interplay between adhesion, consumption and aerotaxis, which allows for an efficient exploration of parameter space and is sufficient to understand the mechanism underlying aggregates.

**Fig. 5:**
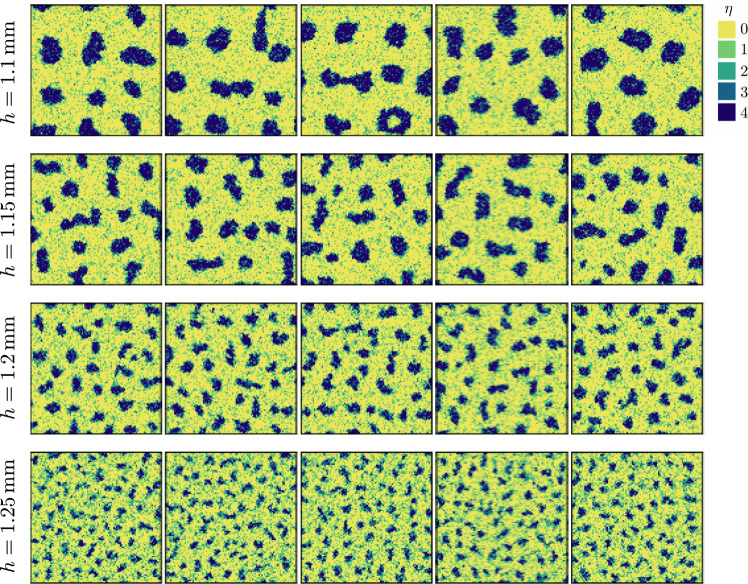
Aggregates in the cell-based simulations for various film height *h*. The average density is $\bar{\rho }=1{0}^{6}$ cm^−2^, the system size $L=100\,\bar{b}=1$ mm, and the simulations involve 10^4^ cells. From top to bottom, the aggregates have typical size *a* = 90, 65, 42, 26 ± 5 μm. From left to right, the simulation time is *t* = 5, 6, 7, 8, 9 × 10^6^ MC steps. The aggregates have reached a steady state but never settle into a static configuration.

*Shape of aggregates*. A series of aggregate configurations are shown in Fig. 5. As regards the shape aggregate, several comments are in order. First, simulated aggregates are not always quasi-circular but can transiently adopt elongated shapes that are rarely seen in experiments. Second, the “gas” of cells outside the domains is rather homogeneous. This is somewhat different from experiments (see Fig. 1) where the largest aggregates are surrounded by domains that are smaller and of lower density. These two differences indicate that our simulations provide only an idealized description of the phenomenon. Third, an aggregate can occasionally be seen with a hole, at least transiently. When too large with respect to its equilibrium size, a domain may nucleate a hole in its center and subsequently break into smaller pieces. While the hole is presumably an artifact of our two-dimensional description, it points to the aggregate interior as the location where the aerotactic effects are the strongest.

*Steady state*. For each film height, two initial conditions were considered: (i) cells are placed at random, or (ii) cells are arranged on a single giant aggregate of circular shape within which $\eta={\eta }_{\max }$. Though the relaxation may require a significant time—several millions of MC steps for the largest aggregates—, the typical aggregate size eventually reaches a plateau independent of the initial conditions, which indicates that a steady state has been attained. Nevertheless, it is clear from Fig. 5 that the aggregates never settle in a static configuration (see also Supplementary Movie 6). Domains continually deform and rearrange, without any trend to further coarsening. This observation shows that domain growth is not kinetically limited and that a genuine steady state with a preferred domain size has been reached. Such a dynamic behavior of domains is also seen in experiments (Supplementary Fig. 2).

### Reporting summary

Further information on research design is available in the Nature Portfolio Reporting Summary linked to this article.

## Supplementary information

Supplementary information

Description for Additional Supplementary Files

Supplementary Movie 1

Supplementary Movie 2

Supplementary Movie 3

Supplementary Movie 4

Supplementary Movie 5

Supplementary Movie 6

Reporting Summary

## Data Availability

The data that support the findings of this study are available from the corresponding authors upon reasonable request. Source data are provided with this paper.

## Source data

Source Data

## Code availability

The codes that support the findings of this study are available from the corresponding authors upon reasonable request.

## References

[1] Gonzalez-Rodriguez D, Guevorkian K, Douezan S, Brochard-Wyart F (2012). Soft matter models of tissues and tumors. Science.

[2] Alert R, Trepat X (2021). Living cells on the move. Phys. Today.

[3] Kang W (2021). A novel jamming phase diagram links tumor invasion to non-equilibrium phase separation. iScience.

[4] Manning ML, Foty RA, Steinberg MS, Schoetz EM (2010). Coaction of intercellular adhesion and cortical tension specifies tissue surface tension. Proc. Natl Acad. Sci. USA.

[5] Canty L, Zarour E, Kashkooli L, François P, Fagotto F (2017). Sorting at embryonic boundaries requires high heterotypic interfacial tension. Nat. Commun..

[6] Angelini TE (2011). Glass-like dynamics of collective cell migration. Proc. Natl Acad. Sci. USA.

[7] Garcia S (2015). Physics of active jamming during collective cellular motion in a monolayer. Proc. Natl Acad. Sci. USA.

[8] Park JA (2015). Unjamming and cell shape in the asthmatic airway epithelium. Nat. Mater..

[9] Sams T, Sneppen K, Jensen MH, Christensen BE, Thrane U (1997). Morphological instabilities in a growing yeast colony: Experiment and theory. Phys. Rev. Lett..

[10] Ben-Jacob E, Cohen I (2000). Cooperative self-organization of microorganisms. Adv. Phys..

[11] Bates F, Fredrickson G (1999). Block copolymers - designer soft materials. Phys. Today.

[12] Leibler L (1980). Theory of microphase separation in block copolymers. Macromolecules.

[13] Fredrickson, G. *The Equilibrium Theory of Inhomogeneous Polymers* (Oxford University Press, 2006).

[14] Seul M, Andelman D (1995). Domain shapes and patterns: the phenomenology of modulated phases. Science.

[15] Möhwald H (1990). Phospholipid and phospholipid-protein monolayers at the air/water interface. Annu. Rev. Phys. Chem..

[16] Seul M, Wolfe R (1992). Evolution of disorder in magnetic stripe domains. I. Transverse instabilities and disclination unbinding in lamellar patterns. Phys. Rev. A.

[17] Huebener RP (1979). Magnetic Flux Structures in Superconductors.

[18] Maclennan J, Seul M (1992). Novel stripe textures in nonchiral hexatic liquid-crystal films. Phys. Rev. Lett..

[19] Bresme F, Oettel M (2007). Nanoparticles at fluid interfaces. J. Phys.: Condens. Matter.

[20] Stradner A (2004). Equilibrium cluster formation in concentrated protein solutions and colloids. Nature.

[21] Banani SF, Lee HO, Hyman AA, Rosen MK (2017). Biomolecular condensates: organizers of cellular biochemistry. Nat. Rev. Mol. Cell Biol..

[22] Hilbert L (2021). Transcription organizes euchromatin via microphase separation. Nat. Commun..

[23] Semenza GL (2012). Molecular mechanisms mediating metastasis of hypoxic breast cancer cells. Trends. Mol. Med..

[24] Scully D (2016). Hypoxia promotes production of neural crest cells in the embryonic head. Development.

[25] Fathollahipour S, Patil PS, Leipzig ND (2018). Oxygen regulation in development: lessons from embryogenesis towards tissue engineering. Cells Tissues Organs.

[26] West CM, van der Wel H, Wang ZA (2007). Prolyl 4-hydroxylase-1 mediates O_2_ signaling during development of Dictyostelium. Development.

[27] Schiavo G, Bisson R (1989). Oxygen influences the subunit structure of cytochrome c oxidase in the slime mold Dictyostelium discoideum. J. Biol. Chem..

[28] Xing, W., Yin, G., Zhang, J. In *Rotating Electrode Methods and Oxygen Reduction Electrocatalysts* (Elsevier, 2014).

[29] Cochet-Escartin O (2021). Hypoxia triggers collective aerotactic migration in dictyostelium discoideum. eLife.

[30] Imperio A, Reatto L (2004). A bidimensional fluid system with competing interactions: Spontaneous and induced pattern formation. J. Phys.: Condens. Matter.

[31] Archer AJ (2008). Two-dimensional fluid with competing interactions exhibiting microphase separation: theory for bulk and interfacial properties. Phys. Rev. E.

[32] Budrene EO, Berg HC (1995). Dynamics of formation of symmetrical patterns by chemotactic bacteria. Nature.

[33] Taktikos J, Lin YT, Stark H, Biais N, Zaburdaev V (2015). Pili-induced clustering of N. Gonorrhoeae Bacteria. PLoS ONE.

[34] Kuan HS, Pönisch W, Jülicher F, Zaburdaev V (2021). Continuum theory of active phase separation in cellular aggregates. Phys. Rev. Lett..

[35] Liu C (2011). Sequential establishment of stripe patterns in an expanding cell population. Science.

[36] Murray J (1989). Mathematical Biology.

[37] Kessin, R. H. *Dictyostelium: Evolution, Cell Biology, and the Development of Multicellularity* (Cambridge University Press 2001).

[38] Turing A (1952). The chemical basis of morphogenesis. Philos. Trans. R. Soc. Lond. B.

[39] Gierer A, Meinhardt H (1972). A theory of biological pattern formation. Kybernetik.

[40] Beaune G (2018). Spontaneous migration of cellular aggregates from giant keratocytes to running spheroids. Proc. Natl Acad. Sci. USA.

[41] Malet-Engra G (2015). Collective cell motility promotes chemotactic prowess and resistance to chemorepulsion. Curr. Biol..

[42] Taylor BL, Zhulin IB, Johnson MS (1999). Aerotaxis and other energy-sensing behavior in bacteria. Annu. Rev. Microbiol..

[43] Deygas M (2018). Redox regulation of EGFR steers migration of hypoxic mammary cells towards oxygen. Nat. Commun..

[44] Slack J (1993). Embryonic induction. Mech. Dev..

[45] Morales JS, Raspopovic J, Marcon L (2021). From embryos to embryoids: how external signals and self-organization drive embryonic development. Stem Cell Rep..

[46] Cheng JY, Ross CA, Smith HI, Thomas EL (2006). Templated self-assembly of block copolymers: top-down helps bottom-up. Adv. Mater..

[47] Stamati K, Mudera V, Cheema U (2011). Evolution of oxygen utilization in multicellular organisms and implications for cell signalling in tissue engineering. J. Tissue Eng..

[48] Wood, R., Donoghue, P. C., Lenton, T. M., Liu, A. G. & Poulton, S. W. The origin and rise of complex life: Progress requires interdisciplinary integration and hypothesis testing. *Interface Focus* **10,** 20200024 (2020).

[49] Bozdag, G. O., Libby, E., Pineau, R., Reinhard, C. T. & Ratcliff, W. C. Oxygen suppression of macroscopic multicellularity. *Nat. Commun*. **12**, 2838 (2021). 10.1038/s41467-021-23104-0 PMC8121917 33990594

[50] De Gennes, P., Brochard-Wyart, F. & Quéré, D. *Capillarity and Wetting Phenomena: Drops, Bubbles, Pearls, Waves* (Springer Verlag, 2004).

[51] Hirose, S., Rieu, J. P., Cochet-Escartin, O., Anjard, C. & Funamoto, K. The oxygen gradient in hypoxic conditions enhances and guides *Dictyostelium discoideum* migration. *Processes* **10**, 318 (2022).

[52] Detcheverry FA, Pike DQ, Nagpal U, Nealey PF, de Pablo JJ (2009). Theoretically informed coarse grain simulations of block copolymer melts: method and applications. Soft Matter.

