# Supplementary material for: Microphase separation of living cells
Source: Nat Commun. 2023 Feb 13;14:796. doi: 10.1038/s41467-023-36395-2 (PMC9925768; doi:10.1038/s41467-023-36395-2)
Supplement: Supplementary file 1 — Supplementary information [file 41467_2023_36395_MOESM1_ESM.pdf]

Supplementary Information for
"Microphase separation of living cells"

SI-A: Steady state
SI-B: Aggregate structure
SI-C: Aggregate response to a step change in oxygen
SI-D: Oxygen measurements, cell consumption and division
SI-E: Analytical model
SI-F: Additional information on Fig. 1e
SI-G: Supplementary references

A. STEADY STATE

To ascertain that our system has reached a steady state, we considered several quantities: the background phase cell density, the mean aggregate size and the distribution of aggregate size.

Supplementary figure 1A shows a semilog plot of the background cell density ρ_{b} as a function of time for the same medium height $h = 1.5\,\mathrm{mm}$ but starting from different initial cell densities ρ_0. Among the three independent experiments, the red curve corresponds to the experiment displayed in Fig. 1a-c of the main text ($\rho_0 = 7.5\,10^4\ \mathrm{cm}^{-2}$). The doubling time in the exponential growth phase is similar for the three experiments, with a value $8 \pm 1\,\mathrm{h}$. The background cell density after aggregation (indicated by arrows) is constant at about $5 \pm 1\,10^5\,\mathrm{cm}^{-2}$.

The size of aggregate (Supplementary Fig. 1B) also exhibits a plateau reached about 24 h to 36 h after the onset of aggregation. The red curve corresponds again to the experiment of Fig. 1a-c ($h = 1.5\,\mathrm{mm}$). The blue curve corresponds to the experiment of Fig. 1d-f of the main text ($h = 0.85\,\mathrm{mm}$, $\rho_0 = 10^5\,\mathrm{cm}^{-2}$). For the latter, we show in Supplementary Fig. 1C two snapshots of the aggregates at times separated by 10 h. To the naked eye, there is no obvious change in the domain size. This can be confirmed quantitatively by computing the distributions of size and inter-aggregate distance (Supplementary Fig. 1D-E). In both cases, there is no change in the histogram bin where the maximum is reached and the increase of the mean average size is only 5% after 10 h. To a very good approximation, one can consider the aggregate have indeed reached a steady state.

Finally, we also report in Supplementary Fig. 1B two experiments where the liquid film is topped with a thin layer of oil to reduce evaporation but not oxygen availability. The curve plateaus are also consistent with a steady state.

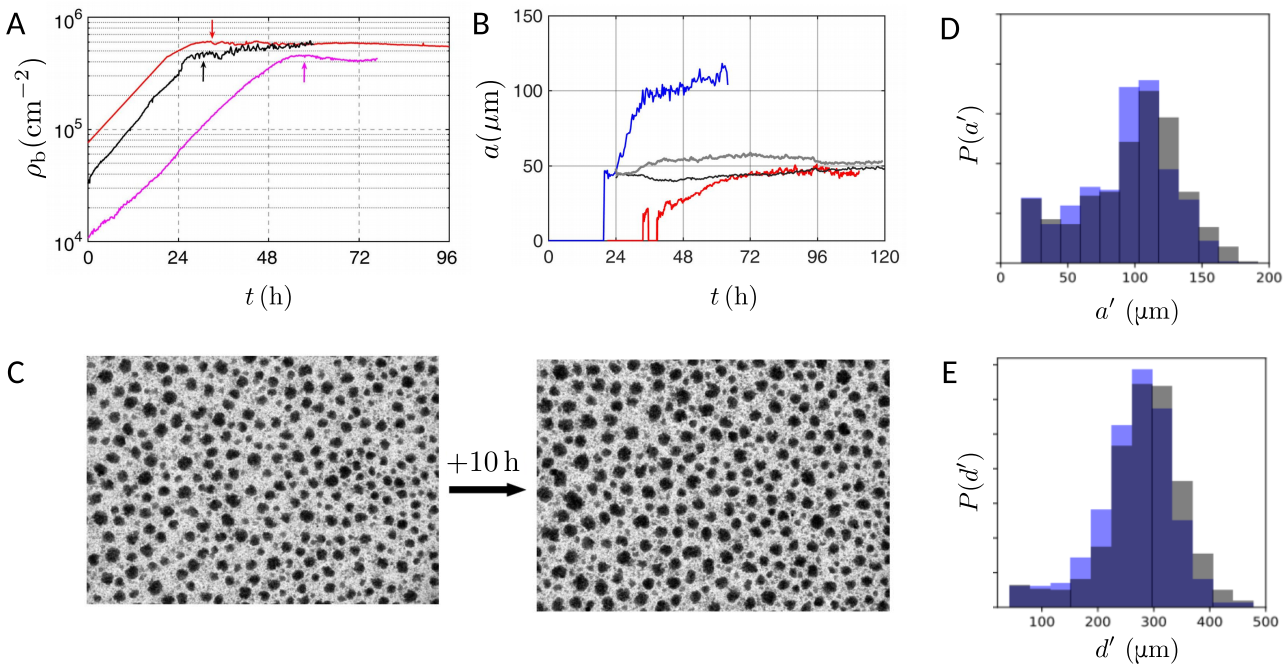

Supplementary Figure 1. (A) Time evolution of the background cell density ρ_{b} The medium height is $h = 1.5\,\mathrm{mm}$ and the init ial cell densitiesare $\rho_0 = 1.2\,10^4$, $2.2\,10^4$ and $7.5\,10^4\ \mathrm{cm}^{-2}$ for pink, black and red curves respectively. (B) Time evolution of the mean aggregate
size a. Blue and red curves: the medium height is $h = 0.85$ and $1.5\,\mathrm{mm}$ respectively. Gray and black curves: two independent experiments where the liquid film is covered with 1.6 ml of paraffin to reduce evaporation, the medium height is $h = 1.5\,\mathrm{mm}$. (C) Snapshots of the experiment corresponding to the blue curve in (B) after $t = 53\,\mathrm{h}$ and 10 h later. (D-E) Superimposed distributions of the domain size $P(a')$ and first neighbor's distance $P(d')$ for the two snapshots at $t = 53\,\mathrm{h}$ (blue) and 10 h later (gray).

Remarkably, aggregates themselves remains very mobile and never settle into a static configuration. This is illustrated in Supplementary Movie 4 and in Supplementary Fig. 2, which shows the trajectory of many aggregates over one day. Aggregates actually move over a distance much larger than the typical inter-aggregate distance, and do so without coalescing or merging with their neighbours.

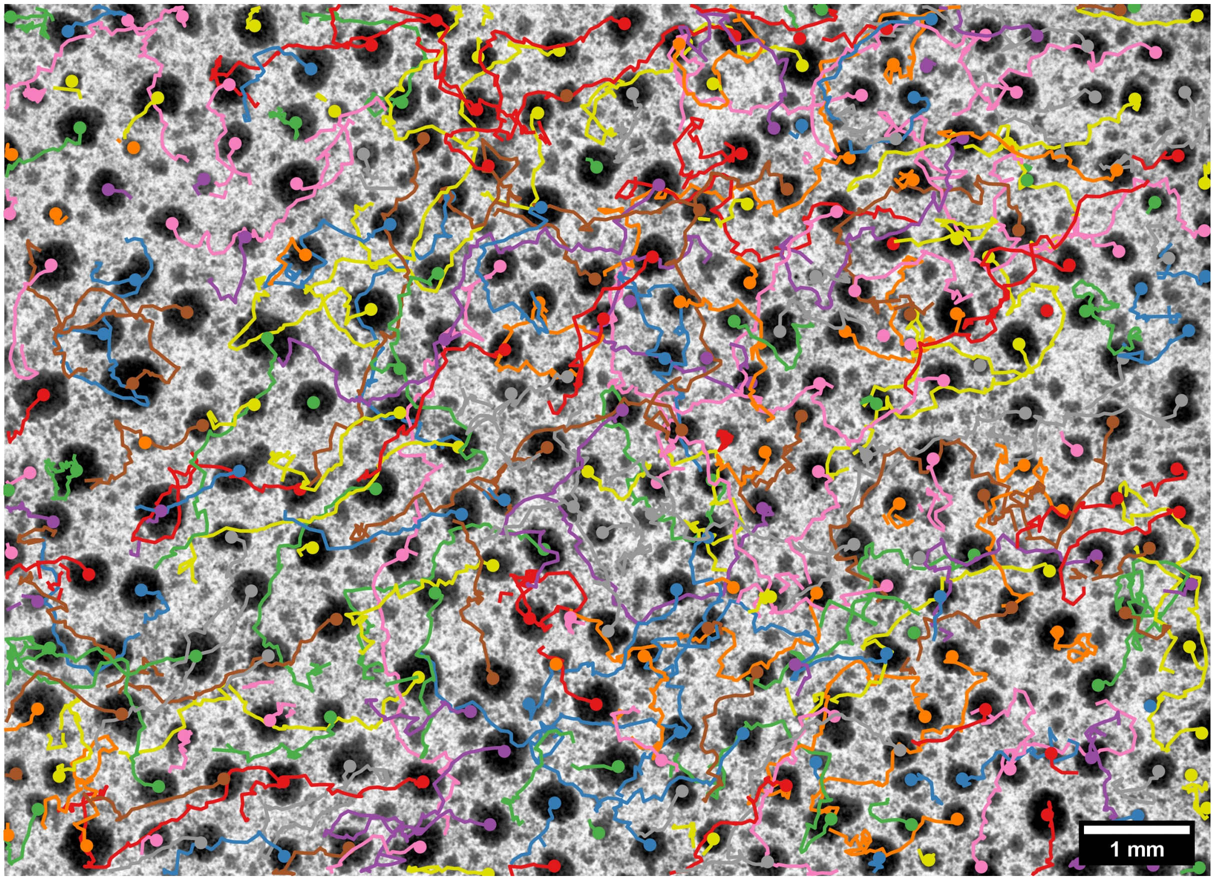

Supplementary Figure 2. Trajectories of domains tracked over 24 h. Each dot indicates the final position. The film height is $h = 0.85$ mm. This experiment was repeated independently two times with similar results.

B. AGGREGATE STRUCTURE

1. *Aggregate cohesion*

Dd cells in development stage in starvation buffers are known to possess three adhesive systems [1]. The first to be expressed is calcium dependent and can be disabled by EDTA (Ethylenediaminetetraacetic acid), a chelator of divalent ions.

Once aggregates reached their steady state, we added 20 µL EDTA at 500 µM in one well of a 6-well plate with about 2 mL of medium. The injection was made drop by drop as gently as possible to avoid any disturbance. The aggregates then start dissociating (Supplementary Movie 3), a process seen immediately in the injection zone and elsewhere in the subsequent hours. These observations indicate that the calcium dependent adhesive system is operative in a nutrient buffer at high cell density and that cell-cell adhesion is responsible for the aggregate cohesion.

2. *Aggregate height*

We first examine a typical aggregate, whose radius 70 µm is close to the typical value $a = 65 \pm 25$ µm obtained with medium height $h = 1.5$ mm. To visualize the three-dimensional structure, we used Z-stacks in transmission mode using 10X objective lens on a confocal (Leica SP5, Germany). The reference slice at $z = 0.00$ µm (Supplementary Fig. 3A) shows the bottom of the aggregate surrounded by a first layer of cells spread on the substrate (yellow arrows) and not very mobile (Supplementary Movie 2). At $z = 7.11$ µm above the reference (Supplementary Fig. 3B), a second layer of cells, more rounded and very mobile is present (red arrows). At $z = 30.81$ µm (Supplementary Fig. 3C), we detect the aggregate upper boundary. Taking into account the uncertainty in the manual estimation of reference level (± 2 µm) and of the upper boundary (± 7 µm), this suggests a height around 30 ± 7 µm.

A second indication of aggregate height is provided by aggregates climbing PDMS vertical pillars, as illustrated in Supplementary Fig. 3D. Most aggregates had a height within 30 µm but aggregates as thick as 40 µm were occasionally encountered.

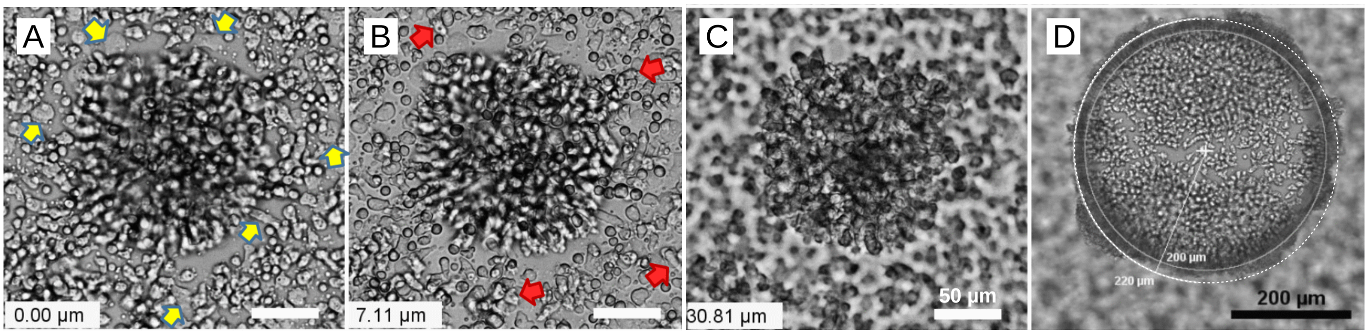

Supplementary Figure 3. Multilayered structure of a typical aggregate (70 µm in radius, medium height $h = 1.5$ mm). (A-C) Confocal slicesat (A) 0.00 µm, (B) +7.11 µm and (C) +30.81 µm. Yellow arrows indicate some cells of the first layer (not mobile) and red
arrows indicate cells of the second layer (rounded and mobile). (D) Side view of aggregates climbing PDMS vertical pillars. The pillar is 200 µm in radius, as shown by the inner circle. The outer circle plotted is at distance 20 µm from the inner circle. The dark layer surrounding the inner circle gives a side view of aggregates. This experiment was repeated independently once with similar results.

3. *Aggregate cell density*

To estimate the projected cell density in aggregates ρ_a, we use the relation $\bar{\rho} = \phi\rho_\mathrm{a} + (1-\phi)\rho_\mathrm{b}$. We consider the typical system shown in Fig. 1a-c and Fig. 2d with $h = 1.5$ mm of medium. From the measured mean cell density $\bar{\rho} = 0.8\,10^6\,\mathrm{cm}^{-2}$ the background cell density $\rho_\mathrm{b} = 0.5\,10^6\,\mathrm{cm}^{-2}$and the surface fraction $\phi = 0.2$, one finds $\rho_\mathrm{a} \simeq 2\,10^6\,\mathrm{cm}^{-2}$. Because of large error bars, the actual value may actually fall in large range around this average, but this provides at least a reasonable estimate.

C. AGGREGATE RESPONSE TO A STEP CHANGE IN OXYGEN

We show in Supplementary Fig. 4 the effect of increasing the amount of oxygen available to cells. Images are separated by 5 hours, a time interval during which cell division is negligible (see Note below). Whatever the initial state fixed by the medium height h – large or small aggregates –, the typical size of domains substantially increases.

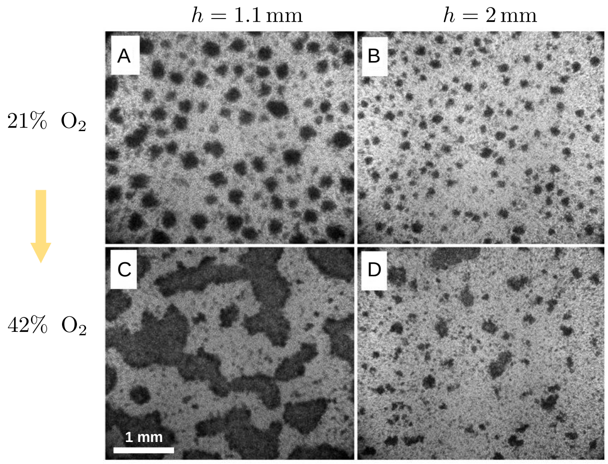

Supplementary figure 4. Effect of a step increase of oxygen on the aggregates. The oxygen level in the atmosphere above the liquid medium isdoubled with respect to normal conditions. (A-B) Bright field images of aggregates formed under normal atmosphere and twomedium heights. (C-D) Images taken 5 h after the step change. This experiment was repeated once with similar results.

We illustrate in Supplementary Fig. 5 the effect of returning to normal oxygen level, after a temporary increase of 3.5 h during which, as above, cell division can be neglected. Though the characteristic radius of aggregates has roughly doubled at high oxygen level, aggregates recover their smaller initial size when going back to normal atmosphere. Such a decrease in size is a strong clue that aggregates are not governed by coarsening. Thus, whether being made artificially smaller as in Fig. 2e or larger as in Supplementary Fig. 5, aggregates return to a preferred domain size, which is a key feature of microphase separation.

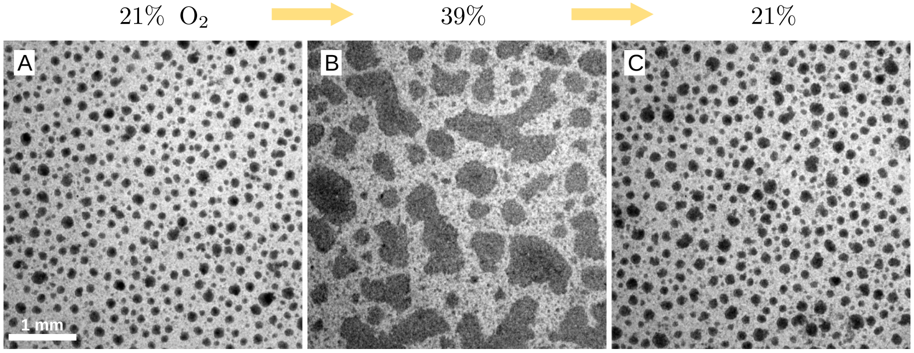

Supplementary figure 5. Effect of a rectangular pulse of oxygen. For time between 61 h and 64.5 h, the oxygen level is nearly doubled withrespect to normal conditions. (A-C) Brightfield images of aggregates formed before (60 h), during (64 h) and after (74 h) thepulse. The medium height is $h = 1.5\,\mathrm{mm}$.

Note : In the initial state with 21% oxygen level, cells have stopped dividing because of severe hypoxic conditions near the substrate. Upon doubling the oxygen level in the atmosphere and increasing oxygen availability, cells might resume their division. However, we showed in Supplementary Fig. 7A that once quiescent, cells need a time lag of 6 hours before they can divide again. Cell division is thus negligible in the process of Supplementary Figs. 4 and 5.

D. OXYGEN MEASUREMENTS, CELL CONSUMPTION AND DIVISION

1. *Oxygen measurements*

Oxygen concentration were obtained using a commercial optical "robust oxygen probe" (OXROB3) coupled to its oxymeter (Firesting, Pyroscience, Aachen, Germany). The measurement is based on the luminescence quenching by oxygen of a redflash indicator deposited at the probe tip.

We systematically controlled the oxygen content in our homemade environmental chamber. More specifically, we measured the gaseous volume percentage, defined as $p_{O_2}/p_{atm} \times 100\%$, where p_{O_2} and p_{atm} are respectively the partial pressure of oxygen and the barometric pressure of ambient air.

To obtain the oxygen concentration at saturation in the HL5 *Dd* growth medium under normal atmosphere, the probe was plunged in 6-mL bottle filled with the medium but no cells. At temperature 22°C , we found $c_s = 250 \pm 20\,\mu$M, in agreement with literature values [2].

2. *Consumption of individual cells*

To measure the oxygen consumption of cells, we filled a 6-mL glass bottle with a concentrated cell suspension, which contains typically $2\,10^7$ cells, and closed it with a cap through which the oxygen probe was plunged in the liquid. The cap was carefully sealed around the probe to avoid external oxygen entering the bottle. During the time required to prepared the cell suspension and the oxygen probe, the oxygen concentration inside the bottle dropped to about 200 µM. Then it linearly decreased to nearly zero in about 1600 s, as shown in Supplementary Fig. 6A. The cell oxygen consumption which is proportional to the slope of this curve is nearly constant (Supplementary Fig. 6B), with a value of $q = 4.2 \pm 0.8\,10^{-17}\,\mathrm{mol\,s^{-1}\,cell^{-1}}$ (average over six experiments), in a concentration range extending from 150 µM down to a few µMs. For lower concentration, the consumption drops abruptly. The typical concentration at which cell consumption becomes concentration dependent is thus in the range $2.5 - 10\,\mu$M, which corresponds to c_{csm}/c_s in the range $[0.01, 0.04]$.

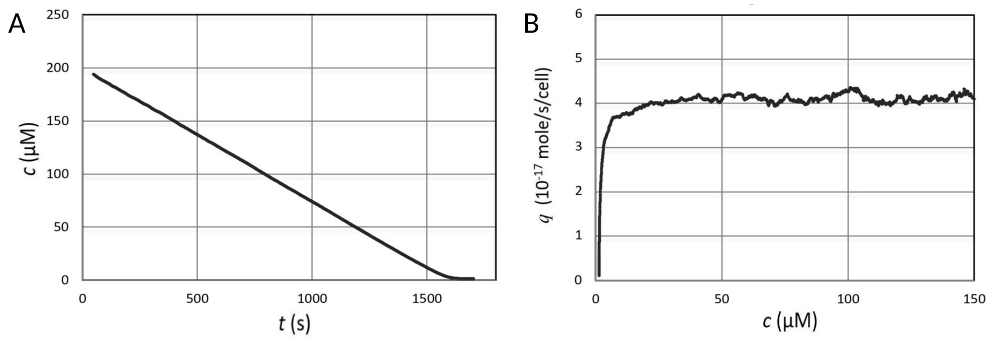

Supplementary Figure 6. Measurement of oxygen consumption by AX2 cells for a typical experiment. (A) Change in oxygen co ncentration as afunction of time due to cell consumption. (B) Deduced cell consumption as a function of the oxygen concentr ation.

3. *Cell division at low oxygen concentration*

Starting from very low cell densities at about $3000\,\mathrm{cm^{-2}}$, we monitored the cell growth by timelapse microscopy under various atmosphere oxygen levels in our environmental chamber. Given the low cell densities which make total consumption small, the dissolved oxygen concentration in the culture medium is simply fixed by the oxygen level outside. Supplementary figure 7A shows a typical growth at two very different oxygen levels. In the initial atmospheric reference condition (21% O_2, $c = c_s = 250\,\mu$M), the growth is exponential with division time $T_{div} \simeq 8$ h (i.e. 4-fold increase during 16 h), in agreement with previous measurements [3] From 22 h to 45 h, pure N_2 was injected in our chamber up, leading to a residual level of 0.15% O_2 ($c = 1.8\,\mu$M). During the first 6 hours of this severe hypoxic condition, the population continues to grow although at a decreasing rate. The cell number then reaches a plateau and eventually slightly decreases (the reason is that because some cells detach and round up, their optical contrast changes and they are partially undetected by the FindMaxima tool of ImageJ). Upon re-injection of 21% O_2 air

at time 45 h, the population still slightly decreases during about 6 h, but it later recovers and grows again. These observations indicate that at $c = 1.8\,\mu$M, cells finish their life cycle before entering a resting phase (quiescence). The existence of the 6 hours lag period upon air re-injection implies that after quiescence, cells need time to resume their normal life cycle.

We tested a range of oxygen levels and observed that the growth index, that is the fold change in the number of cells between between $t = 0$ h and $t = 16$ h, is changing with oxygen concentration (Supplementary Fig. 7B). In particular, at $c = 5\,\mu$M and $c = 12.5\,\mu$M, the growth index is $N(16\,\text{h})/N(0\,\text{h}) = 1.2$ and 2 respectively. Our values (squares) are in agreement with literature values [4,5] for the AX3 cell lines (triangles and circles respectively). Interpolating the data with a simple exponential form (dotted line) leads to the estimate $c_{\text{div}} \simeq 10\,\mu$M for the concentration at which the growth is divided by two with respect to the reference in normoxic conditions. Accordingly, we use $c_{\text{div}}/c_{\text{s}} = 0.05$ in Equation (1) of the main text.

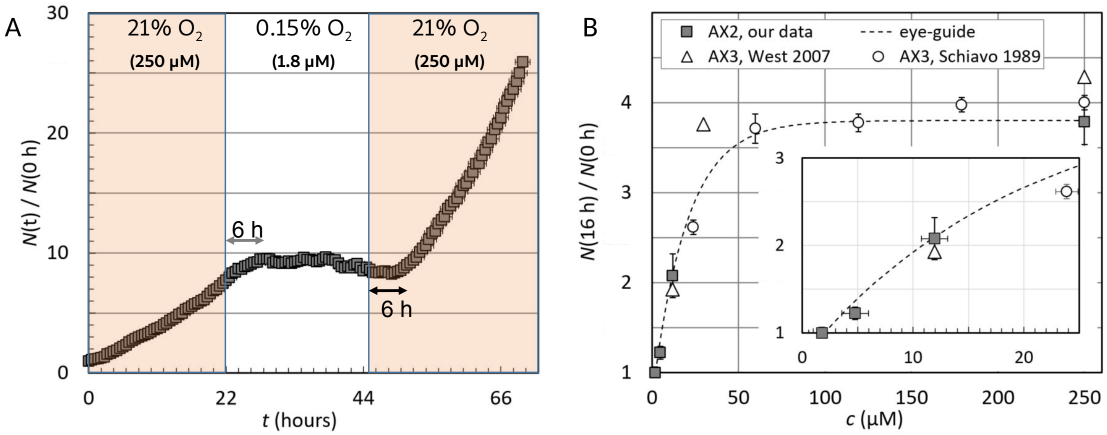

Supplementary Figure 7. Oxygen dependence of cell growth. (A) Number of cells $N(t)$ as a function of time, with changing oxygen levels.(B) Dependence of growth index on oxygen concentration in culture medium. The growth index is the fold change in thenumber of cells between $t = 0$ h and $t = 16$ h. Filled squares show our measurements with the AX2 cell line (mean+/-SD,
number of independent sample measurement is 3, 4, 3 and 7 for $c = 1.8, 4.8, 11.9$ and 250 µM respectively). Triangles and circles correspond to literature value with AX3 cell lines [4,5] interpolated from 24 h to 16 h assuming an exponential growth. Error bars were taken from original articles. The inset shows the $0 - 25\,\mu$M region. The dotted line is a guide for the eye.

E. ANALYTICAL MODEL

1. Model definition

Our simplified model for aggregate is shown in Supplementary Fig. 8. An aggregate is represented as a disk of zero thickness and radius a placed on the bottom surface. The cell density is ρ_a within the aggregate and ρ_b in the background outside. Because the mean cell density $\bar{\rho}$ will be fixed in the following, an aggregate can grow in size only by depleting the surrounding region. The individual cell consumption q is assumed constant. The oxygen flux consumed by the cells is $J_\mathrm{a} = q\rho_\mathrm{a}$ and $J_\mathrm{b} = q\rho_\mathrm{b}$ on the aggregate and background respectively. The aggregate is not isolated but surrounded by identical neighbours arranged on a hexagonal array. To make the calculation analytically tractable, the hexagonal boundary of the unit cell is replaced by a disk of radius b of equal area, so that azimuthal invariance is recovered. A vanishing lateral flux is imposed at this effective boundary. The concentration on the top surface is fixed to the saturation value c_s. The surface fraction of aggregate is $\phi = a^2/b^2$ and the mean density $\bar{\rho} = \phi\rho_\mathrm{a} + (1-\phi)\rho_\mathrm{b}$. We will assume that the aggregate cell density ρ_a is a fixed value, independent of aggregate size and surface fraction.

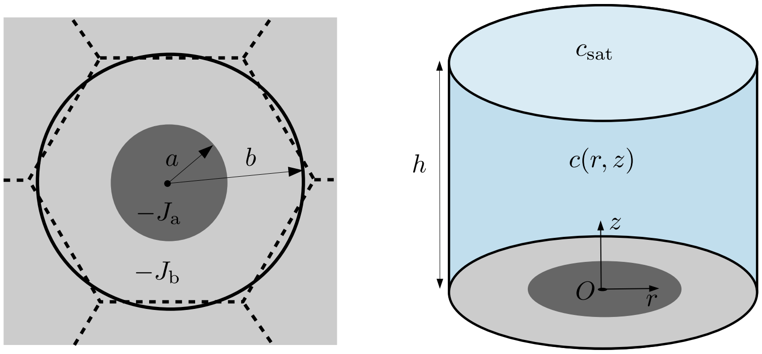

Supplementary Figure 8. Configuration considered in the analytical model: an aggregate is represented as a disk of radius a, below a liquidfilm of height h. The flux of oxygen consumed is J_a on the aggregate and J_b on the background outside. We compute the
concentration at the origin and seek for which aggregate radius a it reaches the target concentration $\hat{c}$.

2. Derivation of analytical solution

Here we give the details of the derivation for the oxygen concentration field $c(r,z)$, which is a purely diffusive problem. The notations are shown in Supplementary Fig. 8. It is convenient to work with the function

$$f(r,z) \equiv 1 - \frac{c(r,z)}{c_\mathrm{s}}. \tag{1}$$

Unless otherwise mentioned, we chose units so that h, D and c_s are all unity. The equation and boundary conditions to be satisfied by $f(r,z)$ are

$$\Delta f = 0, \qquad 0 < r < b,\ \ 0 < z < h, \tag{2a}$$
$$\partial_r f = 0, \qquad r = b, \tag{2b}$$
$$\partial_z f = \mathcal{J}(r), \qquad z = 0, \tag{2c}$$
$$f = 0, \qquad z = 1, \tag{2d}$$

where $\Delta = r^{-1}\partial_r(r\partial_r) + \partial^2_{zz}$ denotes the Laplacian. The flux perpendicular to the lateral wall is taken as zero and $\mathcal{J}(r)$ is the arbitrary flux imposed on the bottom surface. Applying classical methods [6], the solution is

$$f(r,z) = A_1(1-z) + \sum_{m=2}^{\infty} A_m J_0(\mu_m r/b)\frac{\sinh(\mu_m(1-z)/b)}{\cosh(\mu_m/b)}, \tag{3a}$$

$$A_1 = \frac{2}{b^2}\int_0^b \mathcal{J}(r) r dr, \qquad A_m = \frac{2}{\mu_m^2 J_0^2(\mu_m)}\int_0^b J_0(\mu_m r/b)\mathcal{J}(r) r dr. \tag{3b}$$

Here, J_n is the Bessel function of order n, μ_m is the mth zero of $J_0' = -J_1$, with the convention $\mu_1 = 0$. We consider an imposed flux

$$-\mathcal{J}(r) = J_{\text{b}} + J_{\text{ab}} H(a-r), \tag{4}$$

with H the Heaviside function, $J_{\text{ab}} \equiv J_{\text{a}} - J_{\text{b}}$ and $\bar{J} = \phi J_{\text{a}} + (1-\phi) J_{\text{b}}$ is the mean flux. We then find for the value at the bottom surface

$$f(r,0) = \bar{J} + a J_{\text{ab}} \Psi(r,a,b), \qquad \Psi(r,a,b) \equiv \sum_{m=2}^{\infty} \frac{2 J_1(\mu_m a/b) J_0(\mu_m r/b)}{\mu_m^2 J_0^2(\mu_m)} \tanh(\mu_m/b). \tag{5}$$

Now, putting back dimensions, the condition that the minimal concentration, obtained at $r = 0$ and $z = 0$, is the target value $\hat{c}$ can be rewritten as

$$\frac{a}{h}\, \psi\left(\frac{a}{b}, \frac{h}{b}\right) = \frac{\xi j_{\text{m}}(h) - \bar{J}}{J_{\text{a}} - \bar{J}}, \qquad \text{with} \;\; \psi(\zeta,\lambda) = \frac{2}{1-\kappa^2} \sum_{m=2}^{\infty} \frac{J_1(\mu_m \zeta) \tanh(\mu_m \lambda)}{\mu_m^2 J_0^2(\mu_m)}, \tag{6}$$

where we used $J_{\text{ab}} = (J_{\text{a}} - \bar{J})/(1-(a/b)^2)$. Since fluxes are proportional to cell densities, Equation (6) gives back Eq. (2), where $\sqrt{\phi} = a/b$ is assumed fixed.

The function $\psi(\zeta,\lambda)$, computed numerically, is plotted in Supplementary Fig. 9. For $\lambda = h/b > 1$, the dependence on λ is negligible and $\psi(\zeta,\lambda)$ can be approximated as $\psi(\zeta,\infty)$. Taking ζ in the range $[0.1, 1]$ includes all surface fraction of interest since $\phi = \zeta^2$. In this domain, the function $\psi(\zeta,\infty)$ has limited variation, being confined in the interval $[0.38 - 0.94]$ and remains a prefactor of order one. Note that this conclusion holds in most of the relevant parameter space but not in the vicinity of h_{min}. In this region, the domain size a diverges while $\psi(\zeta, \lambda = \sqrt{\phi}\, h/a)$ approaches zero as $\lambda \to 0$.

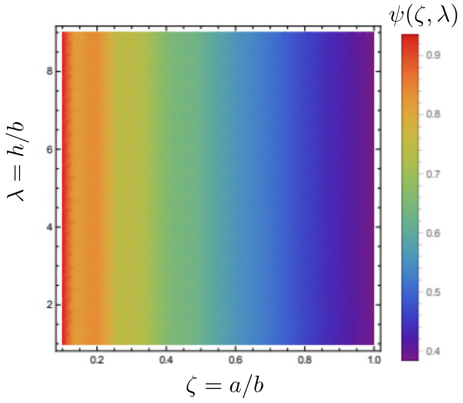

Supplementary Figure 9. Function $\psi(\zeta,\lambda)$ defined in Eq. (6).

3. *Choice of parameters*

Here we explain the parameters taken in applying the model. The values for c_{s}, D and q were introduced in the main text. The saturation concentration $c_{\text{s}} = 250\,\mu\text{M}$ and the cell consumption rate $q = 4.2\,10^{-17}\,\text{mol}\,\text{s}^{-1}$, were both measured experimentally as detailed in Section D of Supplementary Information, The diffusion coefficient is $D = 2\,10^{-5}\,\text{cm}^2\,\text{s}^{-1}$. For the aggregate (projected) cell density, a reasonable estimate is $\rho_{\text{a}} = 2\,10^6\,\text{cm}^{-2}$ (Section B of Supplementary Information) which corresponds to $h_{\text{min}} = 0.55\,\text{mm}$ according to Eq. (3). As regards the critical concentration $\hat{c}$, the steady state we consider has constant cell number because cells have stopped dividing (Supplementary Fig. 1A), which suggests a concentration below c_{div} everywhere, and a significantly lower value at the aggregate center where it is minimal. We therefore fix $\hat{c}/c_{\text{s}} = 0.01$ and note that this value could be doubled or halved with comparable results. Finally, the surface fraction ϕ remains a free parameter. It is bounded by the maximal value $\phi_{\text{max}} = \bar{\rho}/\rho_{\text{a}}$ reached when all cells are inside aggregates and none outside (empty background with $\rho_{\text{b}} = 0$). It turns out that the choice of ϕ between ϕ_{max} and lower values ($0.16\,\phi_{\text{max}}$ for instance in Fig. 3e) has only a limited influence on the results, as visible in Fig. 3e.

F. ADDITIONAL INFORMATION ON FIG. 1e

For each point of Fig. 1e, identified by color and film thickness h, we report in Supplementary Table 1 the mean number of aggregates by frame ($\mathcal{N}_{\mathrm{agg,frame}}$) as well as the number of frames used to obtain the point (N_{frame}).

Color point	h (mm)	$\mathcal{N}_{\mathrm{agg,frame}}$	N_{frame}
Blue	1.1	85	6
Blue	1.62	124	6
Blue	2.03	128	6
Blue	2.66	103	6
Orange	0.89	53	6
Orange	1.51	114	6
Orange	1.62	75	6
Orange	2.66	144	6
Red	0.89	97	6
Red	1.1	122	6
Red	1.93	164	6
Green	0.944	45	1
Green	1.2	39	1
Green	1.46	53	1
Green	1.72	60	1
Purple	1.25	48	1
Purple	1.54	63	1
Purple	1.69	65	1
Purple	2.12	50	1
Purple	2.41	41	1
Purple	2.89	0	1

Supplementary Table 1. Additional information on Fig. 1e. For each point, we indicate the mean number of aggregates by fr ame and thenumber of frames used to obtain the point. Time interval between frames is 20 min.

G. SUPPLEMENTARY REFERENCES

[1] Chen, G., Xu, X., Thomson, A., Yang, C., Regulation of spatiotemporal expression of cell-cell adhesion molecules during development of Dictyostelium discoideum. *Dev. Growth Differ.* **53**, 518 (2011).

[2] Xing, W., Yin, G., Zhang, J., Oxygen solubility, diffusion coefficient, and solution viscosity, in *Rotating Electrode Methods and Oxygen Reduction Electrocatalysts*, (Elsevier 2014).

[3] d'Alessandro, J., Mas, L., Aubry, L., Rieu, J. P., Rivière, C., Anjard, C., Collective regulation of cell motility using an accurate density-sensing system. *J. R. Soc. Interface*, **15**, 20180006 (2018).

[4] West, C. M., van der Wel, H., Wang, Z. A., Prolyl 4-hydroxylase-1 mediates O_2 signaling during development of Dictyostelium. *Development* **134**, 3349 (2007).

[5] Schiavo, G., Bisson, R., Oxygen influences the subunit structure of cytochrome c oxidase in the slime mold Dictyostelium discoideum. *J. Biol. Chem.* **264**, 7129 (1989)

[6] Carslaw, H. & Jaeger, J. *Conduction of heat in solids.* (Oxford University Press, 1959).
