# Supplementary material for: Microphase separation of living cells
Source: Nat Commun. 2023 Feb 13;14:796. doi: 10.1038/s41467-023-36395-2 (PMC9925768; doi:10.1038/s41467-023-36395-2)
Supplement: Supplementary file 2 — Description for Additional Supplementary Files [file 41467_2023_36395_MOESM2_ESM.pdf]

SUPPLEMENTARY MOVIES

Supplementary Movie 1: Microphase separation in *Dictyostelium discoideum* under submerged conditions. Cells were plated at $t = 0$ at $7.5\,10^4$ cell/cm^2 (not shown). Cells first grow exponentially and at time $t = 24$ h (first image of the movie), the density is close to $4.5\,10^5$ cell/cm^2. At $t = 35$ h, when the density reached $7.5\,10^5$ cell/cm^2, cells start to gather on the right side, forming small transient loose aggregates. At $t = 60$ h, the full field of view is invaded by aggregates. These aggregates grow in a very dynamic way: they exchange single cells with the surrounding and fuse with other clusters to become bigger. Occasionally, they fully melt or divide in a fission event. Between 60 h and 114 h, almost 2.5 days, they move continuously but never agglomerate into a giant aggregate. Microscope settings: X4 objective lens, confocal in transmission mode. Time label is in hours. Bar is 500 µm.

Supplementary Movie 2: Higher magnification, one hour sequence, of an aggregate 2.5 days after entering in the microphase-separated state. The aggregate lies on a first carpet of flattened single cells in close contact with the substrate. These flattened cells on the substrate layer are poorly mobile and still isolated; they do not form a dense monolayer. The aggregate is moving by exchanging a few cells with the gas phase but it keeps most of its constituent cells as well, displaying large deformation and apparent traction from its periphery. The focus plane on this movie is around the background of cells and the inner part of the aggregate appears partially transparent. Microscope settings: X10 objective lens, confocal in transmission mode. Time label is min:sec, bar is 50 µm.

Supplementary Movie 3: Effect of EDTA on aggregate size. Aggregates were formed for 2.5 days in normoxic conditions (21% O_2). The EDTA was added at time $t = 0$, which is the first frame of the movie. Microscope settings: X4 objective, inverted microscope in transmission mode. Time label is h:min, bar is 1000 µm.

Supplementary Movie 4: Dynamics of aggregates in the steady microphase-separated state. Large field of view ($8.33 \times 6.25\,\mathrm{mm}^2$) of the aggregate displacements between $t = 40$ h and $t = 64$ h after plating the cells. The medium height is $h = 1.5$ mm. Microscope settings: X1 objective lens, Leica MZ16 binocular equipped with a TL3000 Ergo transmitted LED light base. Time label is min:sec, bar is 50 µm.

Supplementary Movie 5: Effect of changing the atmosphere oxygen level on aggregate size. Aggregates were formed for 2.5 days in normoxic conditions ($\sim$21% O_2) before taking the first image. Right after the first image referred as $t = 0$, the oxygen level was decreased to 10% by injecting N_2, before returning to 21% at $t =$1:40 (h:min). After the descending step, the aggregates slowly shrink and break in small aggregates. After the ascending step, the aggregates slowly regrow. Microscope settings: X4 objective, inverted microscope in transmission mode. Time label is h:min, bar is 200 µm.

Supplementary Movie 6: Simulated aggregates in steady state. The system has size $L = 100\,\bar{b} = 1$ mm, density $\bar{\rho} = 10^6\,\mathrm{cm}^{-2}$ and includes 10^4 cells. With film height $h = 1.15$ mm, the typical aggregate size is $a = 65$ µm. To reach steady state, $5\,10^6$ MC steps were performed before the movie starts. The total duration shown is $T = 2\,10^5$ MC steps.
