# Supplementary material for: Microphase separation of living cells
Source: Nat Commun. 2023 Feb 13;14:796. doi: 10.1038/s41467-023-36395-2 (PMC9925768; doi:10.1038/s41467-023-36395-2)
Supplement: Supplementary file 9 — Reporting Summary [file 41467_2023_36395_MOESM9_ESM.pdf]

nature portfolio

Corresponding author(s): Jean-Paul RIEU

Last updated by author(s): Jan 6, 2023

Reporting Summary

Nature Portfolio wishes to improve the reproducibility of the work that we publish. This form provides structure for consistency and transparency in reporting. For further information on Nature Portfolio policies, see our Editorial Policies and the Editorial Policy Checklist.

Statistics

For all statistical analyses, confirm that the following items are present in the figure legend, table legend, main text, or Methods section.

n/a	Confirmed	
☐	☒	The exact sample size (*n*) for each experimental group/condition, given as a discrete number and unit of measurement
☐	☒	A statement on whether measurements were taken from distinct samples or whether the same sample was measured repeatedly
☒	☐	The statistical test(s) used AND whether they are one- or two-sided<br>*Only common tests should be described solely by name; describe more complex techniques in the Methods section.*
☒	☐	A description of all covariates tested
☒	☐	A description of any assumptions or corrections, such as tests of normality and adjustment for multiple comparisons
☐	☒	A full description of the statistical parameters including central tendency (e.g. means) or other basic estimates (e.g. regression coefficient) AND variation (e.g. standard deviation) or associated estimates of uncertainty (e.g. confidence intervals)
☒	☐	For null hypothesis testing, the test statistic (e.g. *F*, *t*, *r*) with confidence intervals, effect sizes, degrees of freedom and *P* value noted<br>*Give P values as exact values whenever suitable.*
☒	☐	For Bayesian analysis, information on the choice of priors and Markov chain Monte Carlo settings
☒	☐	For hierarchical and complex designs, identification of the appropriate level for tests and full reporting of outcomes
☒	☐	Estimates of effect sizes (e.g. Cohen's *d*, Pearson's *r*), indicating how they were calculated

Our web collection on statistics for biologists contains articles on many of the points above.

Software and code

Policy information about availability of computer code

Data collection	Micromanager 2.0 gamma (Open Source Software for Control and Automation of Microscope Hardware), LAS software 3.4 (software for control of Leica SP5 confocal microscope), Leica LAS X for binocular 3.4.2
Data analysis	Python 3.9.10 (custom), ImageJ (custom code), C

For manuscripts utilizing custom algorithms or software that are central to the research but not yet described in published literature, software must be made available to editors and reviewers. We strongly encourage code deposition in a community repository (e.g. GitHub). See the Nature Portfolio guidelines for submitting code & software for further information.

Data

Policy information about availability of data

All manuscripts must include a data availability statement. This statement should provide the following information, where applicable:

- Accession codes, unique identifiers, or web links for publicly available datasets
- A description of any restrictions on data availability
- For clinical datasets or third party data, please ensure that the statement adheres to our policy

A data availability statement was included. Source data are provided. The code that supports the findings of this study are available from the corresponding authors upon reasonable request.

Human research participants

Policy information about studies involving human research participants and Sex and Gender in Research.

Reporting on sex and gender	n/a
Population characteristics	n/a
Recruitment	n/a
Ethics oversight	n/a

Note that full information on the approval of the study protocol must also be provided in the manuscript.

Field-specific reporting

Please select the one below that is the best fit for your research. If you are not sure, read the appropriate sections before making your selection.

[x] Life sciences   [ ] Behavioural & social sciences   [ ] Ecological, evolutionary & environmental sciences

For a reference copy of the document with all sections, see nature.com/documents/nr-reporting-summary-flat.pdf

Life sciences study design

All studies must disclose on these points even when the disclosure is negative.

Sample size	Sample size was not predetermined. Experiments of Fig. 2e-d consist in detecting the most probable aggregate size or cell density for a given experimental condition. Each experimental condition gives a unique medium height even if experiments were repeated 5 times. Hence, we did not average experiments. We searched for a compromise between aggregate detection and sample size. The numbers of aggregates detected using the larger microscope field of view could be several hundreds at a given time. This number was enough to ensure a good distribution of size and the possibility to define a clear maximun and a full width at half maximum. A new Table of sample size for Fig. 2e was included in the supplementary information section G. For cell density estimation (Fig. 2d), cell counting was repeated 3 times. Proliferation experiments were repeated 3 to 6 times (supplementary Fig. 7). Cell consumption experiments were repeated 6 times.
Data exclusions	No data exclusion
Replication	In absence of any contamination or technical issue all replication attempts were successful.
Randomization	No randomization was applied. We worked with a single cell line.
Blinding	We did not conduct experiments to compare our cell line with another strain or with the same cell submitted to a particular pharmacological treatment. The only changes were steps of oxygen concentration which have a quick, strong and visible impact on the experiment making not applicable any blind test.

Reporting for specific materials, systems and methods

We require information from authors about some types of materials, experimental systems and methods used in many studies. Here, indicate whether each material, system or method listed is relevant to your study. If you are not sure if a list item applies to your research, read the appropriate section before selecting a response.

Materials & experimental systems

n/a	Involved in the study	
[x]	[ ]	Antibodies
[x]	[ ]	Eukaryotic cell lines
[x]	[ ]	Palaeontology and archaeology
[x]	[ ]	Animals and other organisms
[x]	[ ]	Clinical data
[x]	[ ]	Dual use research of concern

Methods

n/a	Involved in the study	
[x]	[ ]	ChIP-seq
[x]	[ ]	Flow cytometry
[x]	[ ]	MRI-based neuroimaging
